# The lipid droplet assembly complex consists of seipin and four accessory factors in budding yeast

**DOI:** 10.1016/j.jbc.2024.107534

**Published:** 2024-07-07

**Authors:** Chao-Wen Wang, Rey-Huei Chen, Yu-Kai Chen

**Affiliations:** 1 Department of Life Sciences, National Cheng Kung University, Tainan, Taiwan; 2 Institute of Molecular Biology, Academia Sinica, Taipei, Taiwan

**Keywords:** seipin, lipid droplet, ER-LD contact site, LD assembly, perilipin, TurboID

## Abstract

Seipin, a crucial protein for cellular lipid droplet (LD) assembly, oligomerizes at the interface between the endoplasmic reticulum and LDs to facilitate neutral lipid packaging. Using proximity labeling, we identified four proteins—Ldo45, Ldo16, Tgl4, and Pln1—that are recruited to the vicinity of yeast seipin, the Sei1-Ldb16 complex, exclusively when seipin function is intact, hence termed seipin accessory factors. Localization studies identified Tgl4 at the endoplasmic reticulum-LD contact site, in contrast to Ldo45, Ldo16, and Pln1 at the LD surface. Cells with compromised seipin function resulted in uneven distribution of these proteins with aberrant LDs, supporting a central role of seipin in orchestrating their association with the LD. Overexpression of any seipin accessory factor causes LD aggregation and affects a subset of LD protein distribution, highlighting the importance of their stoichiometry. Although single factor mutations show minor LD morphology changes, the combined mutations have additive effects. Lastly, we present evidence that seipin accessory factors assemble and interact with seipin in the absence of neutral lipids and undergo dynamical rearrangements during LD formation induction, with Ldo45 acting as a central hub recruiting other factors to interact with the seipin complex.

The lipid droplet (LD) is a ubiquitous and evolutionarily conserved organelle present in nearly all organisms. Structurally, it comprises a neutral lipid core encased by a phospholipid monolayer with selected proteins on its surface. Functionally, the LD plays a pivotal role in cellular fat management and is instrumental in maintaining cellular lipid and membrane homeostasis. Anomalies in cellular LDs are frequently observed in pathological conditions associated with prevalent diseases, including obesity, diabetes, atherosclerosis, and cancer. Given the intricate interplay between lipids and diseases, it is important to understand the dynamics, biogenesis, growth, and maintenance of LDs.

Cellular LDs form from the endoplasmic reticulum (ER), where two primary types of neutral lipids—triacylglycerols (TAGs) and sterol esters—are synthesized through the catalytic activities of neutral lipid synthesizing enzymes. These enzymes include diacylglycerol acyltransferases (DGAT1 and DGAT2 in humans, and Dga1 and Lro1 in yeast) responsible for TAG synthesis, and sterol acyltransferases (ACATs in humans, and Are1 and Are2 in yeast) involved in sterol ester production. Neutral lipids that accumulate in the ER coalesce to create neutral lipid lenses, expanding in size and ultimately bud toward the cytoplasmic leaflet of the ER, giving rise to nascent LDs (1). These LDs undergo further growth, developing into mature LDs with a unique proteome.

In most cell types, the homologs of the human lipodystrophy protein seipin play a crucial role in controlling the size of LDs and maintaining lipid homeostasis (2, 3, 4, 5, 6). Several recent studies further indicate that seipin defines the initiation site of LD formation at the ER-LD contact site (7, 8). Structural data reveal that seipin forms a ring-shaped oligomer composed of 10 to 12 closely intertwined seipin subunits (9, 10, 11). The luminal domain of seipin comprises a long hydrophobic helix embedded within the ER inner leaflet for TAG concentration. Additionally, two beta sheets in the luminal domain are folded into a hydrophobic pocket, resembling a lipid-binding domain, potentially mediating interactions with lipids. In contrast to human and fly seipin, the luminal domain of the seipin homolog Sei1 in yeast lacks a hydrophobic central helix and the ability to concentrate TAG (11). Thereby, it requires Ldb16 to concentrate TAG within the Sei1 ring by providing critical hydrophobic residues within its transmembrane helices.

Human seipin is thought to work with lipid droplet assembly factor 1 (LDAF1) /promethin to facilitate TAG filling into the LD core (12). In yeast, Ldo45 is the homolog of human LDAF1, and the protein is made by overlapping ORF with a smaller protein Ldo16 (13, 14). Besides seipin and LDAF1, a number of proteins are found at the ER-LD contact site and are thought to be involved in LD biogenesis or connecting LDs with the ER (15, 16, 17). Interestingly, these specific ER-LD contact sites are sequentially loaded with proteins implicated in LD biogenesis before the actual initiation of LD formation (18). Altogether, a seipin-associated machinery is likely involved in ensuring a consistent framework for the selective sorting of lipids and proteins during the orchestration of LD biogenesis and assembly.

To investigate the precise machinery of seipin, we established a proximity labeling assay for identifying seipin's intimate neighbors. This assay unveiled four proteins—Ldo45, Ldo16, Tgl4, and Pln1—in close proximity to the yeast seipin, Sei1-Ldb16 complex. Subcellular localization studies indicate that Tgl4 is enriched at the ER-LD contact site together with seipin, which is distinct from Ldo45, Ldo16, and Pln1 that are targeted to the LD surface. Compromised seipin function results in association of these proteins predominantly with supersized but not clustered LDs. We further show that overexpressing these proteins affects LD morphology and protein targeting, while their deletions lead to abnormal LD morphology. Notably, we observed alteration of these proteins during the packaging of neutral lipids, aligning with the concept that LD assembly is orchestrated by a precisely regulated molecular machine. Collectively, we name the four proteins seipin accessory factors and propose that they form the LD assembly complex with seipin at the ER-LD contact site, exerting modulatory effects on LD assembly.

## Results

### Ldo45 and Ldo16 are brought into close proximity to seipin

**Figure 1:**
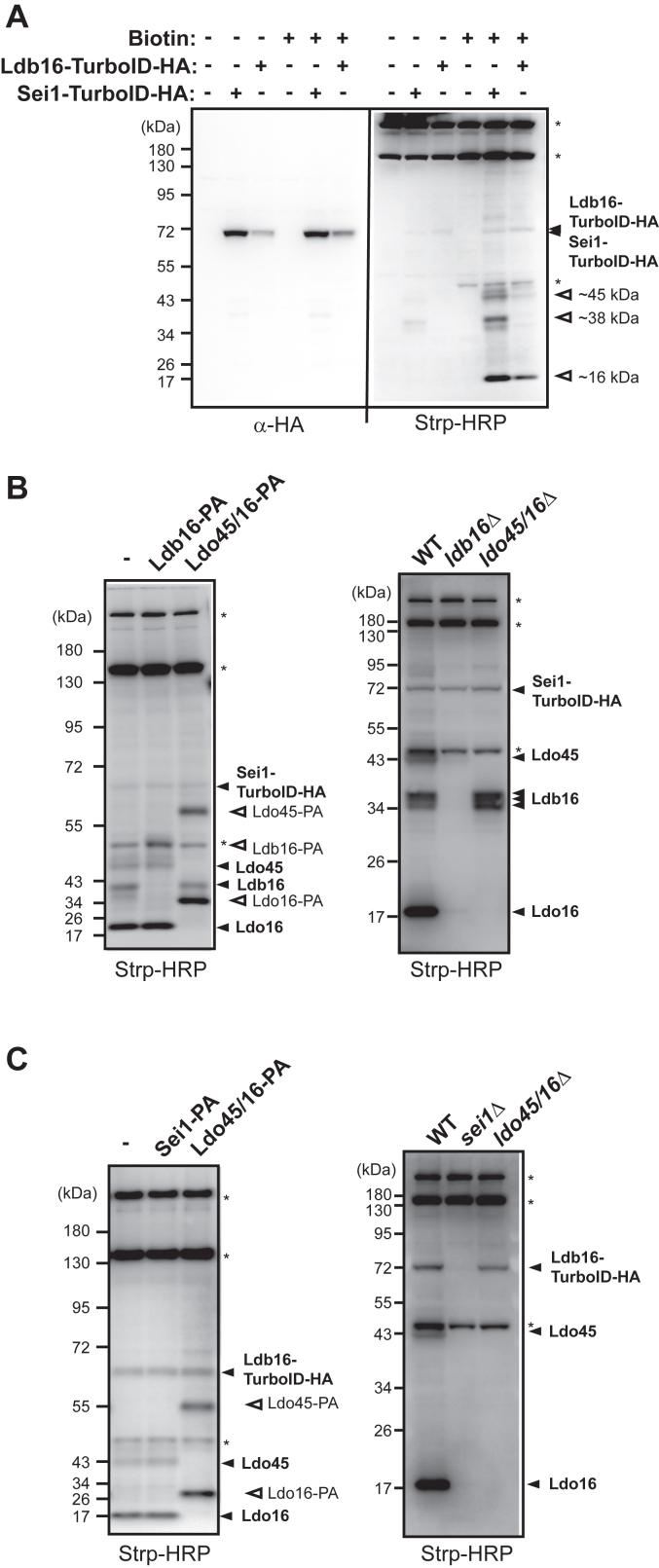
**Sei1-TurboID-HA and Ldb16-TurboID-HA labeling identified Ldo45 and Ldo16 as proximity neighbors**. *A*, yeast strains CWY12910, 12931, and 12912, harboring Sei1-TurboID-HA (+) or Sei1 (−) and Ldb16-TurboID-HA (+) or Ldb16 (−), were cultured at 30 °C and subsequently treated with (+) or without (−) biotin for 6 h. Protein extracts from the cell were prepared and analyzed by Western blotting with an anti-HA antibody and with a Strp-HRP conjugated antibody to detect biotin. The same membrane blot was cut for the Western blotting procedure, allowing for the comparison of protein sizes. *B*, (*left*) the yeast strain harboring Sei1-TurboID-HA (CWY12931) was compared with the strain harboring Sei1-TurboID-HA and Ldb16-PA (CWY12991) or Ldo45/16-PA (CWY12993) for biotin labeling. (*right*) the Sei1-TurboID-HA strain (CWY12931) was compared with the Sei1-TurboID-HA strain lacking Ldb16 (CWY12975) or Ldo proteins (CWY12948) for biotin labeling. Samples were analyzed by Western blotting using Strp-HRP. (∗), nonspecific bands detected by Strp-HRP. *C*, (*left*) the yeast strain harboring Ldb16-TurboID-HA (CWY12912) was compared with the strain harboring Ldb16-TurboID-HA and Sei1-PA (CWY12986) or Ldo45/16-PA (CWY12989) for biotin labeling. (*right*) the Ldb16-TurboID-HA strain (CWY12912) was compared with the Ldb16-TurboID-HA strain lacking Sei1 (CWY12951) or Ldo proteins (CWY12954) for biotin labeling. ∗, nonspecific bands detected by Strp-HRP. HA, hemagglutinin; HRP, horseradish peroxidase.

To explore the precise machinery associated with seipin, we established a simple and highly reproducible TurboID proximity labeling assay using the yeast strain lacking the major biotinylated protein Arc1. This method facilitated the identification of proteins proximal to seipin through Western blotting with a streptavidin (Strp)-horseradish peroxidase (HRP) conjugate. Importantly, seipin tagged with TurboID-hemagglutinin (HA) in the *arc1Δ* strain exhibits normal LDs, indicating its functionality (Fig. S1). In a standard reaction involving 50 μM biotin labeling for 6 h, Sei1-TurboID-HA, but not Sei1 alone, revealed three major bands at estimated molecular weights of ∼16 kD, ∼38 kD, and ∼45 kD (Fig. 1*A*). When the TurboID-HA tag was attached to the other yeast seipin subunit, Ldb16, two major proteins of ∼16 kD and ∼45 kD were labeled. In both cases, Strp-HRP also detected proteins running at the sizes of Sei1-TurboID-HA (Sei1 is ∼32 kD and TurboID-HA is ∼35 kD, thus a band at ∼67 kD is expected for Sei1-TurboID-HA) and Ldb16-TurboID-HA (Ldb16 runs at ∼38 kD on SDS-PAGE, thus a band at ∼72 kD is expected for Ldb16-TurboID-HA), albeit with weaker signals compared to the major bands aforementioned (Fig. 1*A*). Given that Sei1 and Ldb16 proteins organize into an oligomeric protein complex for neutral lipid clustering, self-biotinylation of Sei1-TurboID-HA and Ldb16-TurboID-HA is anticipated.

To identify the specific proteins biotinylated by seipin, we introduced protein A (PA) tags to potential candidates at their chromosomal locus in the Sei1-TurboID-HA strain for the standard biotinylation assay (Fig. 1*B*, left). Adding a PA tag to the candidate protein will increase its molecular weight by ∼12 kD. If the candidate protein is indeed biotinylated, we would anticipate the emergence of a new band corresponding to the addition of the 12 kD moiety, replacing the original candidate protein size in the Strp-HRP blots. When the standard Sei1-TurboID-HA biotinylation assay was conducted in the strain expressing Ldb16-PA, a noticeable shift of the ∼38 kD protein to the size of Ldb16-PA at ∼50 kD was observed, indicating that the ∼38 kD protein is Ldb16 (Fig. 1*B*, left). The Ldo45 and Ldo16 are proteins of 45 kD and 16 kD previously found to be associated with seipin (13, 14). When the standard Sei1-TurboID-HA biotinylation assay was conducted in the strain expressing Ldo16-PA, the ∼45 kD and ∼16 kD bands disappeared, being replaced by two higher molecular weight bands, while the ∼38kD Ldb16 signal remained unchanged (Fig. 1*B*, left). Given that Ldo45 and Ldo16 are expressed through overlapping ORFs and, after splicing, the carboxyl terminus of Ldo45 is Ldo16 (13, 14), this result supports that the ∼45 kD and ∼16 kD proteins are Ldo45 and Ldo16, respectively. Consistently, in the Sei1-TurboID-HA strain lacking Ldo45 and Ldo16, the biotinylation results only showed the ∼38 kD Ldb16 signal, without ∼45 kD and ∼16 kD signals (Fig. 1*B*, right). Intriguingly, when the biotinylation assay was performed in the strain with Sei1-TurboID-HA lacking Ldb16, not only did the ∼38 kD Ldb16 signal disappear, but the ∼45 kD and ∼16 kD signals of Ldo proteins were also diminished (Fig. 1*B*, right). Thus, the recruitment of Ldo proteins to seipin necessitates the integrity of seipin, aligning with previous findings (13, 14).

Using a similar approach, we validated the two major biotinylated proteins labeled by Ldb16-TurboID-HA as Ldo45 and Ldo16, based on their size shift when tagged by PA tag and their disappearance when deleted in the Ldb16-TurboID-HA proximity labeling assay (Fig. 1*C*). In *sei1Δ* cells, Ldb16-TurboID-HA became unstable as previously reported (19) and failed to biotinylate Ldo45 and Ldo16 (Fig. 1*C*, right). However, Ldb16-TurboID-HA did not successfully label Sei1. The TurboID proximity labeling assay exhibits constraints, primarily limited to labeling only cytoplasmic parts of integral membrane proteins within ∼10 nm radius and is dependent on the availability of lysine residues in nearby proteins. Given that the main portion of Sei1 is embedded in the ER lumen with its short N and C terminus exposed to the cytoplasm (19), it is possible that Ldb16-TurboID-HA failed to label Sei1 for these reasons. Overall, we conclude that the two yeast Ldo proteins intimately associate with seipin, reminiscence of their counterparts human LDAF1 and seipin, raising a possibility that they are organized into an evolutionarily conserved complex mediating LD assembly.

### Tgl4 and Pln1 associate with Ldo proteins and seipin

Considering the potential limitations of the TurboID labeling assay, we sought to explore whether additional proteins might coexist with Sei1, Ldb16, Ldo45, and Ldo16 in the LD assembly complex. We captured biotinylated proteins labeled by Sei1-TurboID-HA and Ldb16-TurboID-HA using Strp-coupled beads and subjected them to mass spectrometry analysis. From the proteome, we selected proteins that are localized to LDs or previously been implicated in interactions with seipin, and antibodies were then generated against these proteins for validation through Western blotting. Specifically, we aimed to identify less abundant proteins or proteins that are transiently brought to close proximity to seipin through biotinylation by Sei1-TurboID-HA and Ldb16-TurboID-HA, which might not be readily detectable in Strp-HRP blot.

**Figure 2:**
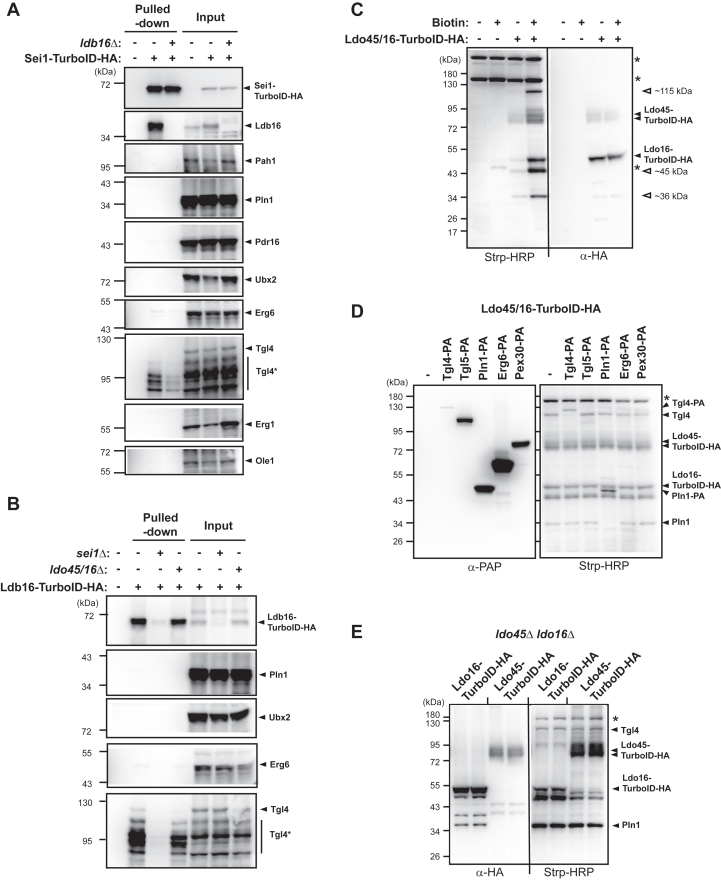
**The identification of Tgl4 and Pln1 proximal to Ldo proteins and seipin.***A*, yeast strains CWY12910, CWY12931, and CWY12975, harboring Sei1-TurboID-HA (+) or Sei1 (−) and with (+) or without (−) Ldb16 deletion, were subjected for biotin labeling for 6 h at 30 °C. Cells were harvested, converted to spheroplasts, and then lysed. The lysates (input) were subjected for Strp pull-down as described in the Experimental procedures. The input and pulled-down fractions were analyzed by Western blotting using an anti-HA antibody and antibodies against the indicated yeast proteins. Tgl4∗, various degraded forms of Tgl4. (*B*) same as (*A*), except that the strains used were CWY12910, CWY12912, and CWY12951, harboring Ldb16-TurboID-HA (+) or Ldb16 (−), with (+) or without (−) additional Sei1 or Ldo protein deletions. *C*, yeast strains CWY12910, and CWY12929, harboring Ldo45/16-TurboID-HA (+) or Ldo45/16 (−), were cultured and subsequently treated with (+) or without (−) biotin for 6 h. Protein extracts from the cell were prepared and analyzed by Western blotting using an anti-HA antibody and Strp-HRP. *D*, the yeast strain CWY12929, CWY13037, CWY13079, CWY12990, CWY13040, and CWY13083 harboring Ldo45/16-TurboID-HA, alone (−) or with additional PA-tagged proteins as indicated, were compared for biotin labeling. Samples were analyzed by Western blotting using a PAP antibody recognizing IgG or Strp-HRP. *E*, the yeast strain CWY13013 lacking two Ldo proteins was transformed with plasmids expressing either Ldo16-TurboID-HA or Ldo45-TurboID-HA. Two independent colonies were cultured in synthetic complete-URA medium at 30 °C and treated with biotin for 6 h. Samples were analyzed by Western blotting using an anti-HA antibody and Strp-HRP. HA, hemagglutinin; HRP, horseradish peroxide; IgG, immunoglobulin G; PAP, peroxidase-anti-peroxidas.

Among proteins tested, we identified Tgl4 as a *bona fide* biotinylated target by either Sei1-TurboID-HA (Fig. 2*A*) or Ldb16-TurboID-HA (Fig. 2*B*). Crucially, the biotinylation of Tgl4 is contingent upon seipin, as Tgl4 signals diminished in the Strp–pulled down fraction of the Sei1-TurboID-HA strain when Ldb16 is deleted, and *vice versa* (Fig. 2, *A* and *B*). Notably, Tgl4 exhibited exceptional instability in cell lysates compared to other proteins examined in Figure 2, *A* and *B*. In fact, it has been demonstrated that Tgl4 has a notably short half-life in WT cells but is stabilized in cells lacking TAG (20). Intriguingly, we observed that the level of biotinylated Tgl4 by Ldb16-TurboID-HA is also reduced in cells lacking Ldo45 and Ldo16, implying a close relationship between Tgl4 and the Ldo proteins (Fig. 2*B*).

During our investigation, we also conducted tests to determine whether Sei1-TurboID-HA biotinylated Tgl4 using the SEY6210 yeast strain background. In this strain, alongside Tgl4, we consistently observed biotinylation of Pln1 by Sei1-TurboID-HA (Fig. S2). As Ldo proteins are major nearest neighbors of Sei1 and Ldb16 (Fig. 1), this raises a possibility that Tgl4 and/or Pln1 might be identified in close proximity to Ldo proteins, in addition to seipin. To explore the relationship between Tgl4 and Ldo proteins, we generated a strain harboring Ldo45/16-TurboID-HA and analyzed their major biotinylated targets with Strp-HRP blots (Fig. 2*C*). Aside from the pronounced self-labeling of Ldo45/16-TurboID-HA, three major bands were detected at estimated sizes of ∼115 kD, ∼45 kD, and ∼36 kD. The ∼115 kD protein corresponds to Tgl4, as evidenced by the size shift of Tgl4-PA reported by Ldo45/16-TurboID-HA (Fig. 2*D*). Intriguingly, Tgl5, the paralog of Tgl4, despite being more abundant and localized to LDs, was not reported by Ldo45/16-TurboID-HA. Additionally, several abundant proteins previously implicated to collaborate with seipin, including Erg6 (18) and Pex30 (15, 16), were also not identified by Ldo45/16-TurboID-HA, Sei1-TurboID-HA, or Ldb16-TurboID-HA in our studies.

Furthermore, we determined that the ∼36 kD protein is Pln1 by the size shift of Pln1-PA reported by Ldo45/16-TurboID-HA (Fig. 2*D*). The observation that either Ldo16-TurboID-HA or Ldo45-TurboID-HA alone labeled both Tgl4 and Pln1 indicates that the association with Tgl4 and Pln1 is a shared feature of the two Ldo proteins (Fig. 2*E*). In summary, our findings unveil the association of Tgl4 and Pln1 with Ldo proteins, which we henceforth refer as the seipin accessory factors. Their proximity to seipin suggests that they may form the LD assembly complex alongside seipin.

### Seipin defines proper localization of seipin accessory factors

**Figure 3:**
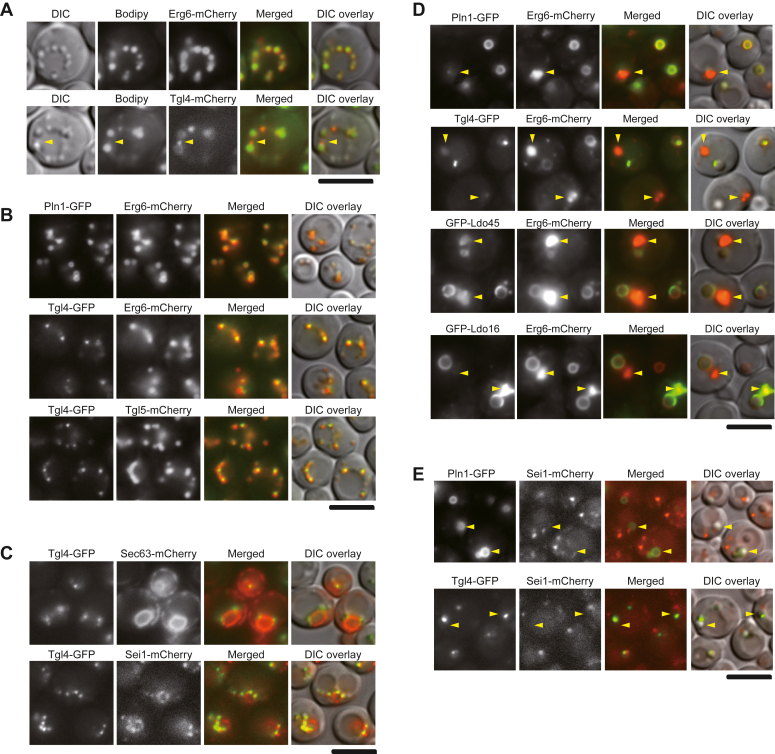
**Proper association of seipin accessory factors with LDs requires functional seipin.***A*, yeast cells harboring Erg6-mCheery (CWY2989) or Tgl4-mCherry (CWY2983) were stained with the neutral lipid dye BODIPY 493/503 and imaged by Olympus IX81 fluorescence microscope. The *yellow arrowhead* denotes punctum of Tgl4-mCherry abutting the LD. The scale bar represents 5 μm. *B*, yeast strains CWY3145, harboring Pln1-GFP and Erg6-mCherry, CWY3127, harboring Tgl4-GFP and Erg6-mCherry, and CWY3223, harboring Tgl4-GFP and Tgl5-mCherry, were imaged by Olympus IX81 fluorescence microscope. The scale bar represents 5 μm. *C*, yeast strain CWY2892, expressing Tgl4-GFP and Sec63-mCherry, and CWY13231, expressing Tgl4-GFP and Sei1-mCherry, were imaged by Olympus IX81 fluorescence microscope. The scale bar represents 5 μm. *D, sei1Δ* yeast strains CWY3240, harboring Pln1-GFP and Erg6-mCherry, CWY3234, harboring Tgl4-GFP and Erg6-mCherry, and the *sei1Δ ldoΔ* strains (CWY13260) harboring Erg6-mCherry and GFP-Ldo45 or GFP-Ldo16 expressed from plasmids were imaged by Olympus IX81 fluorescence microscope. These cells accumulated either supersized or small clustered LDs. The *yellow arrowheads* denote the small clustered LDs. The scale bar represents 5 μm. *E*, *ldb16Δ* cells harboring Sei1-mCherry and Pln1-GFP (CWY13255) or Tgl4-GFP (CWY13249) as indicated were imaged by Olympus IX81 fluorescence microscope. The *yellow arrowheads* denote the supersized LDs. The scale bar represents 5 μm. LD, lipid droplet.

We proceeded to investigate the subcellular localization of the seipin accessory factors. While most of these factors were previously established to associate with LDs, Tgl4 exhibited a distinct localization pattern, particularly noticeable when LDs increased in size in culture post diauxic shift. In contrast to Erg6-mCherry, which enveloped the neutral lipid core stained by BODIPY 493/503, Tgl4-mCherry concentrated as discrete puncta adjacent to the Bodipy signal (Fig. 3*A*). Colocalization studies revealed that, unlike Pln1-GFP, which perfectly overlapped with Erg6-mCherry, the Tgl4-GFP puncta associated with one side of LDs labeled either by Erg6-mCherry or Tgl5-mCherry, indicating an unique subdomain localization (Fig. 3*B*). To gain further insights into the nature of the Tgl4-enriched puncta, we conducted colocalization studies of Tgl4-GFP with Sec63-mCherry and Sei1-mCherry (Fig. 3*C*). The results indicated that the Tgl4-GFP puncta reside within the Sec63-mCherry-labeled ER and exhibited a strong correlation with Sei1-mCherry (Fig. 3*C*). Thus, Tgl4 likely associates with seipin at the ER-LD contact site.

Next, we analyzed the subcellular localization of seipin accessory factors in cells with compromised seipin function, which accumulated aberrant LDs of both supersized and clustered. In contrast to Erg6-mCherry, which unambiguously localized to both pools of LDs, Pln1-GFP exhibited a preference for supersized LDs over clustered LDs (Fig. 3*D*). GFP-Ldo45 and GFP-Ldo16 displayed uneven localization to the two populations of LDs within the same cells. Intriguingly, Tgl4-GFP appeared as discrete puncta adjacent to one side of supersized LDs and was reduced from the clustered LDs in the *sei1Δ* mutant (Fig. 3*D*). In cells lacking Ldb16, Sei1-mCherry localized to puncta with some adjacent to the side of supersized LDs marked by Pln1-GFP (Fig. 3*E*). Interestingly, in the *ldb16Δ* mutant, most of the Tgl4-GFP puncta adjacent supersized LDs did not seem to colocalize with Sei1-mCherry (Fig. 3*E*). Thus, bringing Tgl4 into the same subdomain as seipin appears crucial for maintaining normal LD structures. Importantly, these subcellular localization results align with the biochemical findings presented in Figures 1 and 2, collectively supporting the notion that seipin is indispensable for maintaining the integrity of ER-LD contact site *via* orchestrating the association of these molecules.

### Proper organization of LD assembly complex is essential for maintaining normal LD morphology and ensuring accurate LD protein targeting

**Figure 4:**
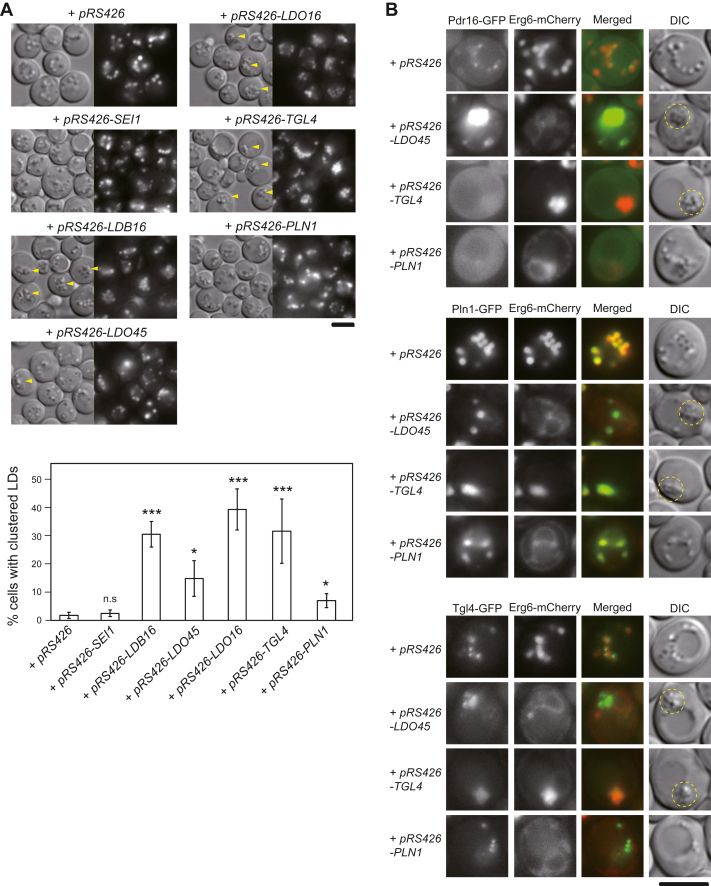
**Overexpression of seipin accessory factors led to aberrant LD morphology and mislocalization of LD proteins**. *A*, WT cells transformed with *pRS426* or *pRS426*-containing *SEI1*, *LDB16*, *LDO45*, *LDO16*, *TGL4*, and *PLN1* were cultured in synthetic complete-URA medium at 30 °C for 24 h. Cells were stained with BODIPY 493/503 and imaged by Olympus IX81 fluorescence microscope. *Yellow arrowheads* denote clustered LDs. The percentage of cells in the population containing clustered LDs was quantified. The *p*-value relative to the control (WT + *pRS426*) is determined by two-tailed Student *t* test. ∗*p* <0.05; ∗∗*p* <0.01; ∗∗∗*p* <0.001.∗. The scale bar represents 5 μm. *B*, the yeast strains expressing Pdr16-GFP and Erg6-mCherry (CWY5802), Pln1-GFP and Erg6-mCherry (CWY3145), and Tgl4-GFP and Erg6-mCherry (CWY3127), respectively, were transformed with *pRS426* or *pRS426*-containing *LDO45*, *TGL4*, and *PLN1* as indicated. Cells were cultured in SC-URA medium at 30 °C for 24 h and imaged by Olympus IX81 fluorescence microscope. Clustered LDs in the cell are circled in *yellow*. The scale bar represents 5 μm. LD, lipid droplet.

Previous studies have demonstrated that overexpression of Ldb16, Ldo45, and Ldo16 results in LD clustering (13, 14). We found that Tgl4 and Pln1 overexpression also induced LD clustering, while Pln1 overexpression generated the phenotype to a small degree (Fig. 4*A*). To further investigate the impact of overexpressing seipin accessory factors within the cell, we performed fluorescence microscopy to assess the distribution of LD proteins. Overexpressing Ldo45 in cells resulted in the extensive localization of Pdr16-GFP onto the clustered LDs (Fig. 4*B*, upper panel), in agreement with that Ldo45 facilitates the targeting of Pdr16 to LDs (13, 14). Concurrently, Erg6-mCherry was excluded from the clustered LDs in the same cell and redistributed back to the ER (Fig. 4*B*, upper panel). Interestingly, a similar redistribution of Erg6-mCherry back to the ER was also observed in Pln1-overexpressing cells, even though LDs in these cells did not aggregate much as they did in cells overexpressing Ldo45 (Fig. 4*B*, upper panel). In addition, Pln1 overexpression also led to the cytosolic localization of Pdr16-GFP, indicating impaired Ldo45 function.

Conversely, Ldo45 overexpression resulted in an uneven distribution of Pln1-GFP, predominantly excluding it from the clustered LDs (Fig. 4*B*, middle panel), reminiscent of Pln1-GFP localization in seipin mutants (Fig. 3*D*). In contrast, Tgl4-GFP was detected on clustered LDs caused by Ldo45 overexpression (Fig. 4*B*, lower panel). Tgl4 overexpression also led to LD clustering. In these cells, Pdr16-GFP was not detected on the clustered LDs, suggesting compromised Ldo45 function (Fig. 4*B*, upper panel). Intriguingly, Pln1-GFP, which did not exhibit a preference for clustered LDs in seipin mutants or in Ldo45-overexpressed cells, target clustered LDs in Tgl4-overexpressing cells (Fig. 4*B*, middle panel). Thus, overexpressing Tgl4 and Ldo proteins likely cause LD clustering *via* distinct mechanisms. Given that Tgl4 and Pln1 overexpression compromised Ldo45 function, these data imply functional synergy among Ldo45, Pln1, and Tgl4 in targeting LD proteins to the LD surface.

**Figure 5:**
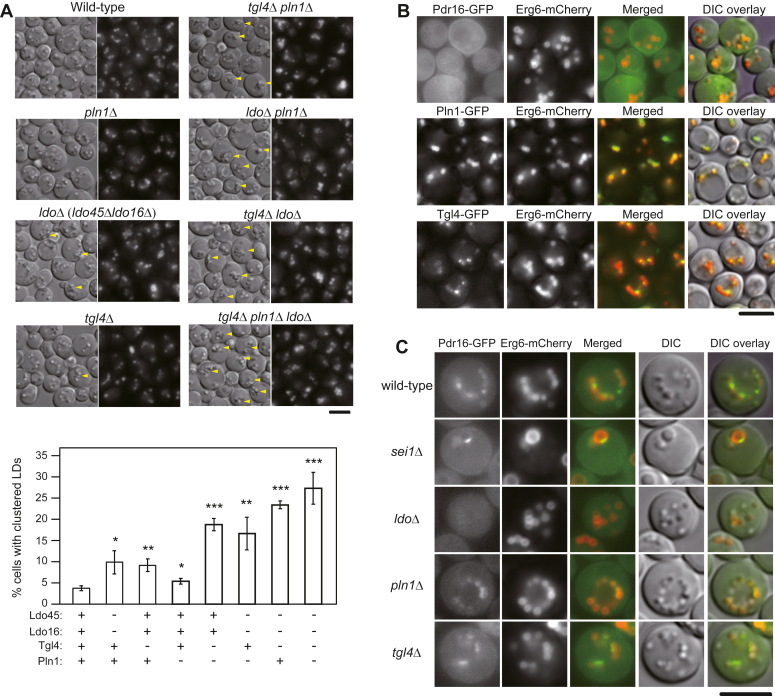
**Combined deletion of seipin accessory factors led to aberrant LD morphology**. *A*, WT (BY4742) and mutant yeast strains CWY13017 (*ldoΔ*), CWY9415 (*tgl4Δ*), CWY3048 (*pln1Δ*), CWY13118 (*pln1Δtgl4Δ*), CWY13151 (*ldoΔpln1Δ*), CWY13109 (*ldoΔtgl4Δ*), and CWY13162 (*ldoΔtgl4Δpln1Δ*) were grown in synthetic-complete medium at 30 °C for 24 h. Cells were stained with BODIPY 493/503 and imaged by Olympus IX81 fluorescence microscope. The *yellow arrowheads* denote clustered LDs. The percentage of cells in the population containing clustered LDs was quantified. The *p*-value relative to the control (WT) is determined by two-tailed Student *t* test. ∗*p* <0.05; ∗∗*p* <0.01; ∗∗∗*p* <0.001. The scale bar represents 5 μm. *B*, *ldoΔ* cells harboring Erg6-mCherry and Pdr16-GFP (CWY13202), Pln1-GFP (CWY13200), or Tgl4-GFP (CWY13208) were grown in synthetic complete medium at 30°C and imaged by Olympus IX81 fluorescence microscope. The scale bar represents 5 μm. *C*, WT strain harboring Pdr16-GFP and Erg6-mCherry (CWY5802) and the corresponding mutant strains deleted of *sei1Δ* (CWY13179), *ldoΔ* (CWY13202), *pln1Δ* (CWY13187), and *tgl4Δ* (CWY13191) were imaged by Olympus IX81 fluorescence microscope. The scale bar represents 5 μm. LD, lipid droplet.

We then investigated whether LD morphology might be affected in cells lacking the seipin accessory factors. Previous studies have indicated that cells deleted of Pln1 reduces TAG production, resulting in slightly fewer LDs (21). Conversely, cells lacking Ldo proteins exhibit slightly larger LDs (13, 14). Similarly, deletion of Tgl4 caused only subtle effects on LDs, showing a slightly fewer and aggregated LD phenotype. Intriguingly, the extent of LD clustering was enhanced in double mutants, such as *tgl4Δ pln1Δ*, and even more pronounced in triple or quadruple mutants, such as *tgl4Δ ldoΔ* and *tgl4Δ pln1Δ ldoΔ* (Fig. 5*A*). Accordingly, we conclude that seipin accessory factors exert functional synergy to control LD morphology in the LD assembly complex.

Deletion of Ldo proteins causes mislocalization of Pdr16 to the cytoplasm, without an effect on the localization of Pln1-GFP to the LD surface or Tgl4-GFP to the puncta adjacent to LDs (Fig. 5*B*). On the other hand, unlike *ldoΔ*, *pln1Δ* or *tgl4Δ* cells did not impair Pdr16 targeting (Fig. 5*C*). Intriguingly, Pdr16-GFP in seipin mutants exhibited as puncta associated with one side of the supersized LD labeled by Erg6-mCherry (Fig. 5*C*), similar to the pattern observed with Tgl4-GFP (Fig. 3*D*). This mislocalization pattern is consistent with the idea that Pdr16 targets LDs through the regulation of the LD assembly complex. As Pdr16 marks a subpopulation of LDs adjacent the vacuole (13), it seems likely that the LD assembly complex comprising seipin and seipin accessory factors is functionally associate with this LD population.

### Alteration of Ldb16 and seipin accessory factors with seipin was observed during LD induction

**Figure 6:**
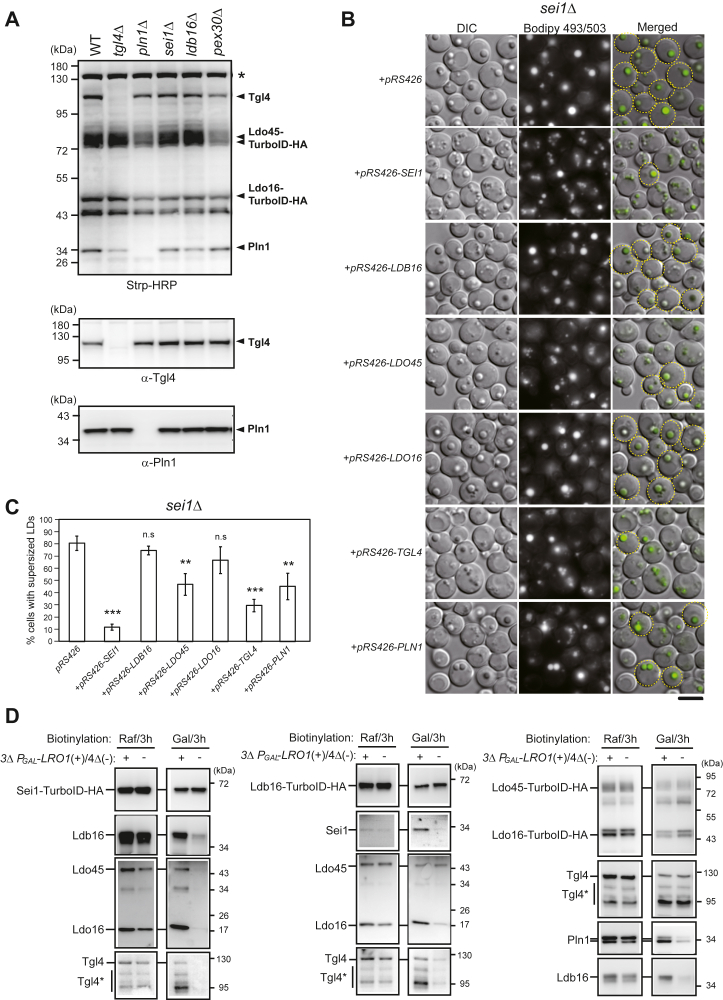
**Dynamics of seipin and seipin accessory factors during LD assembly.***A*, *arc1Δ* (CWY12929), *arc1Δtgl4Δ* (CWY13049), *arc1Δpln1Δ* (CWY13076), *arc1Δsei1Δ* (CWY12957), *arc1Δldb16Δ* (CWY12960), and *arc1Δpex30Δ* (CWY13025) strains expressing Ldo45/16-TurboID-HA were cultured in synthetic-complete medium at 30 °C and treated with biotin for 6 h enabling biotin labeling. Protein extracts from the cell were prepared and analyzed by Western blotting with Strp-HRP, and antibodies against Tgl4 and Pln1. *B*, *sei1Δ* strain (CWY3033) transformed with pRS426 or pRS426-containing *SEI1*, *LDB16*, *LDO45*, *LDO16*, *TGL4*, and *PLN1* vectors were cultured in synthetic complete-URA medium at 30 °C for 24 h. Cells were stained with BODIPY 493/503 and imaged by Olympus IX81 fluorescence microscope. *Yellow circles* denote cells with supersized LDs. The scale bar represents 5 μm. *C*, the percentage of cells in the population containing clustered LDs of data in (*B*) was quantified. The *p*-value relative to the control (*sei1Δ*+pRS426) is determined by two-tailed Student *t* test. ∗*p* <0.05; ∗∗*p* <0.01; ∗∗∗*p* <0.001. *D*, the yeast 3Δ *P*_*GAL*_*-LRO1* Sei1-TurboID-HA strain (+), CWY12322, and the 4Δ Sei1-TurboID-HA strain (−), CWY13167; the 3Δ *P*_*GAL*_*-LRO1* Ldb16-TurboID-HA strain (+), CWY12529, and the 4Δ Ldb16-TurboID-HA strain (−), CWY13102; and the 3Δ *P*_*GAL*_*-LRO1* Ldo45/16-TurboID-HA strain (+), CWY13007, and the 4Δ Ldo45/16-TurboID-HA (−) strains, CWY13264, were cultured at 30 °C and shifted to raffinose (Raf) or galactose (Gal) medium in the presence of 50 μM biotin for 3 h. Cells were harvested, converted to spheroplasts, followed by lysis. The lysates were subjected for Strp pull-down as described in the Experimental procedures and the pulled-down fractions were analyzed by Western blotting with anti-HA, anti-Tgl4, anti-Pln1, anti-Ldo16, and anti-Ldb16 antibodies as indicated. Tgl4∗, various degraded forms of Tgl4. HA, hemagglutinin; HRP, horseradish peroxide; LD, lipid droplet.

We next sought to determine how LD assembly complex forms. Taken advantage of Ldo45/16-TurboID-HA proximity labeling assay, we analyzed association of seipin accessory factor in various strain backgrounds. Ldo45/16-TurboID-HA reported the proximity of Tgl4 in the absence of Pln1 and Pln1 in the absence of Tgl4, respectively (Fig. 6*A*). Moreover, Ldo45/16-TurboID-HA also reported the proximity of Tgl4 and Pln1 in cells lacking Sei1, Ldb16, and Pex30, which suggests that the assembly of seipin accessory factors is independent of seipin. In fact, LDs can form even without seipin. Given that the seipin accessory factors we characterize in this study all associated with supersized LDs in seipin mutants (Fig. 3*D*), we next asked whether perturbation of the stoichiometry of seipin accessory factors might impact supersized LD morphology. Intriguingly, overexpressing Ldo45, Tgl4, and Pln1 in *sei1Δ* cells reduced the fraction of supersized LDs (Fig. 6*B* and quantification in 6*C*). Thus, the synergy and/or activities of seipin accessory factors might impact LD assembly even in the absence of seipin.

We further investigated whether the integrity and dynamics of the seipin accessory factors might be subject to regulation by neutral lipids using the LD induction platform (22). In yeast, neutral lipid synthesis involves Are1, Are2, Dga1, and Lro1. Sei1-TurboID-HA reporter was generated in a quadruple mutant (4Δ), which lacked all four of these enzymes and cannot form LDs, serving as controls for no LDs and for different carbon sources in the experiment. We also generated the 3Δ *P*_*GAL*_*-LRO1* strain in which Are1, Are2, and Dga1 were deleted and the promoter of the residual TAG synthesis enzyme Lro1 was replaced with a *GAL* promoter, allowing for controlled induction of LD formation by galactose addition.

Both the 3Δ *P*_*GAL*_*-LRO1* Sei1-TurboID-HA and 4Δ Sei1-TurboID-HA strains exhibited no LDs when cultured in raffinose medium. In both cases, Sei1-TurboID-HA labeling for 3 h resulted in comparable biotinylation levels of Sei1-TurboID-HA, Ldb16, Ldo45, Ldo16, and Tgl4 as seen in the Strp-pulled-down fractions (Fig. 6*C*, left panel). Thus, seipin and seipin accessory factors already assembled into a complex before the onset of neutral lipid filling. When the biotinylation assay was conducted in cells shifted to galactose medium, the 3Δ *P*_*GAL*_*-LRO1* Sei1-TurboID-HA strain started producing TAG and thus formed LDs (Fig. S3), while the 4Δ Sei1-TurboID-HA strain was unable to form LDs. The proximity labeling assay was carried out within 3 h, the time when numerous tiny LDs emerged in the 3Δ *P*_*GAL*_*-LRO1* Sei1-TurboID-HA strain (Fig. S3). Sei1-TurboID-HA labeling in galactose medium showed stronger signals of Ldb16, Ldo45, Ldo16, and Tgl4 in the LD-forming 3Δ *P*_*GAL*_*-LRO1* strain compared to the LD-lacking 4Δ strains (Fig. 6*D*, left panel). As the elevated biotinylation activities may be attributed to a closer proximity of TurboID with target molecules or a conformational change, the data suggest that Ldb16 and seipin accessory factors dynamically interact with Sei1 during the early phase of LD assembly.

We also performed Ldb16-TurboID-HA and Ldo45/16-TurboID-HA labeling in the LD-inducible and LD-deficient strains to gain further insights. Our data revealed that Ldb16-TurboID-HA in the 3Δ *P*_*GAL*_*-LRO1* strain, relative to the 4Δ strain, reported greater levels of Sei1, Ldo16, and Tgl4 upon galactose but not raffinose treatments, reflecting TAG-triggered dynamics (Fig. 6*D*, middle panel). By contrast, the biotinylation level of Ldb16-TurboID-HA and Ldo45 reported by Ldb16-TurboID-HA remained unchanged (Fig. 6*D*, middle panel), which may suggest a unique association of Ldo45 with Ldb16 during LD assembly. Moreover, Ldo45/16-TurboID-HA reported similar levels of Ldo proteins, Tgl4, Pln1, and Ldb16 in proximity in raffinose conditions (Fig. 6*D*, right panel), consistent with that the assembly of seipin accessory factors and the interaction of seipin accessory factors with seipin is independent of TAG. Upon galactose induction, Ldo45/16-TurboID-HA reported an increased level of Pln1 and Ldb16, but not Tgl4, in the LD-forming 3Δ *P*_*GAL*_*-LRO1* strain compared to the LD-lacking 4Δ strains (Fig. 6*D*, right panel). Altogether, these data indicate alterations in the LD assembly complex that occur during LD formation, likely reflecting structural changes depending on the availability of neutral lipids. Importantly, these alterations reinforce functional synergy among the seipin accessory factors and seipin proteins during LD assembly.

**Figure 7:**
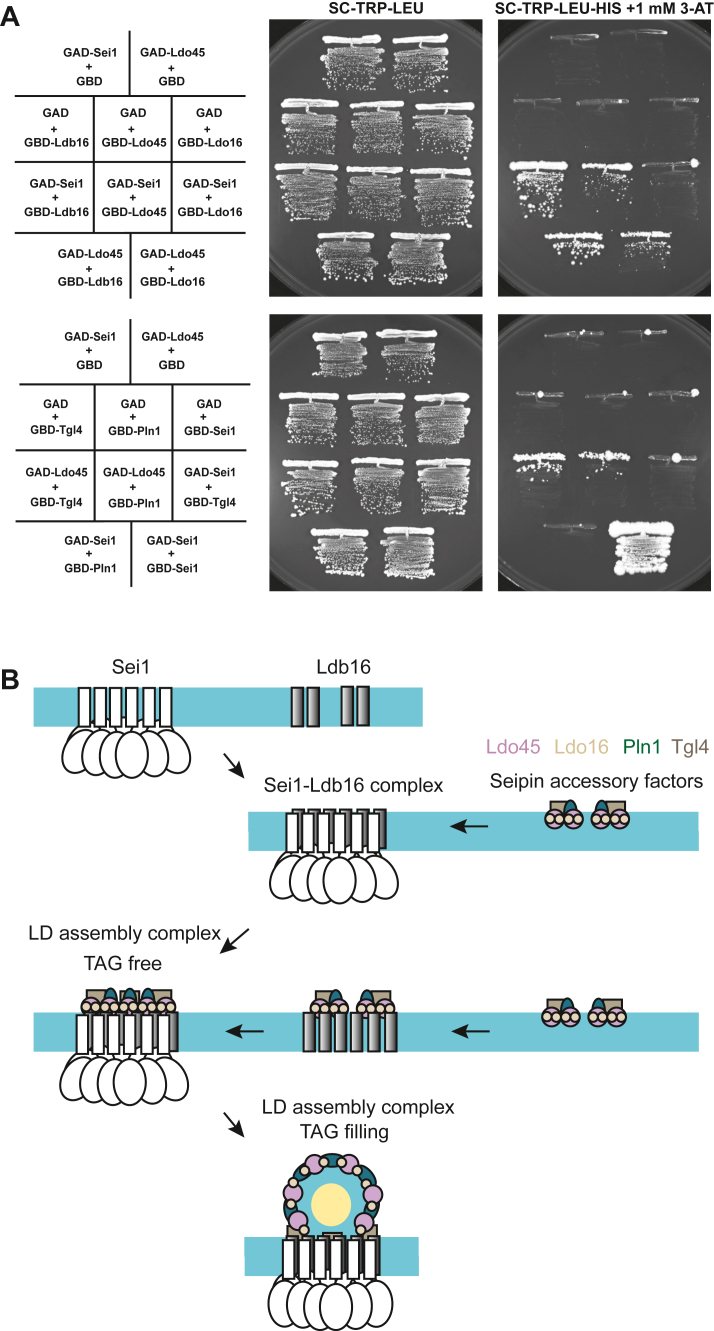
**Model of LD assembly in the budding yeast**. *A*, PJ69-4a cells expressing Gal4 activation domain (AD) and binding domain (BD) alone or fused with Sei1, Ldb16, Ldo45, Ldo16, Tgl4, or Pln1 from plasmids as indicated were streaked on SCD-LEU-TRP or SCD-LEU-TRP-HIS plates containing 1 mM 3-AT and photographed. *B*, the model illustrating the sequential events leading to forming LD assembly complex in yeast. The Sei1 and Ldb16 proteins initiate the assembly of the yeast seipin complex. The complex further incorporates four seipin accessory factors—Ldo45, Ldo16, Pln1, and Tgl4—that interact with each other to form a subcomplex and is recruited to the yeast seipin complex through interacting with seipin, culminating in the establishment of a functional LD assembly complex. Notably, the LD assembly complex can form even in the absence of neutral lipids, but it dynamically rearranges when neutral lipids are present. As neutral lipids accumulate, Ldo45, Ldo16, and Pln1 components are propelled toward the developing LD membrane, while the majority of Tgl4 maintains its residence within the ER subdomain along with the seipin complex. This orchestrated movement contributes to the functional organization of the LD assembly complex in coordination with lipid and protein sorting. ER, endoplasmic reticulum; LD, lipid droplet.

To gain further insights into the interplay among seipin accessory factors and their interactions with seipin, we conducted yeast two-hybrid analyses for all LD assembly complex molecules shown in this study. We found that Ldo45 confers two-hybrid interactions with Ldo16, Pln1, and Tgl4 (Fig. 7*A*), which raises the possibility that Ldo45 acts as the hub of seipin accessory factors. Moreover, yeast two-hybrid assays revealed an interaction between Ldo45 and Ldb16, as well as a weaker interaction between Ldo45 and Sei1. Except Ldo45, we did not identify two-hybrid interactions of Sei1 or Ldb16 with the other three seipin accessory factor in our analysis. Collectively, we propose a model as illustrated in Figure 7*B* that seipin accessory factors assemble into a subcomplex with Ldo45 acting as a hub recruiting other seipin accessory factors to interact with the seipin complex, which is important for the structural integrity of LDs.

## Discussion

The ER-derived cytoplasmic LDs play a key role in storing excess metabolic energy in the form of neutral lipids in most types of cells. The formation of LDs is shown to involve the evolutionarily conserved protein seipin, although the mechanistic details remain enigmatic. By our analysis of nearest neighbors to seipin subunits in yeast, we present compelling evidence that two key proteins, Ldo45 and Ldo16, are prominently brought into the proximity of seipin exclusively when seipin complex Sei1-Ldb16 is intact (Fig. 1). We further identify Tgl4 and Pln1 as major targets for proximity labeling by Ldo proteins, which also associate with intact seipin (Fig. 2). Accordingly, we designate Ldo45, Ldo16, Tgl4, and Pln1 as seipin accessory factors.

To understand the role of these seipin accessory factors in LD assembly, we scrutinize their subcellular localization, investigate the LD phenotypes, and analyze their interaction dynamics. We identify that Tgl4 predominantly localizes at the ER-LD contact site, while Ldo45, Ldo16, and Pln1 reside on the monolayer surface of larger LDs (Fig. 3, *A*–*C*). These factors may work together to direct LD formation at the interface between ER and LDs. We also discovered that Tgl4 appears as discrete puncta adjacent to supersized LDs, while Ldo45, Ldo16, and Pln1 remain on the surface of supersized LDs, under conditions where seipin function is compromised (Fig. 3*D*). In the pool of small clustered LDs within the same cell, the association of Tgl4 and Pln1 is evidently diminished, and a similar phenomenon is occasionally observed with Ldo45 and Ldo16 (Fig. 3*C*). This raises the interesting possibility that supersized LDs formed in cells lacking functional seipin may be attributed to the activities of seipin accessory factors. In support of this notion, we found that the association of seipin accessory factors remains even in cells lacking seipin (Fig. 6*A*) and that altering the association of seipin accessory factors through the overexpression of these individual components readily reduces the presence of supersized LDs in cells lacking seipin (Fig. 6, *B* and *C*).

LD aggregation is commonly observed in seipin mutants. We explored the impact of increased expression levels of each of the seipin accessory factors and found overexpressing Tgl4 and Pln1, similar to Ldo45 and Ldo16 as reported previously, led to LD aggregation (Fig. 4*A*). Through analyzing LD protein targeting, we observed that increased Pln1 expression, akin to Ldo45, prevented Erg6 targeting to the LD surface (Fig. 4*B*). However, targeting of Pdr16 to the LD requires Ldo45, but not Pln1 and Tgl4 (Fig. 5*B*). Rather, Pln1 and Tgl4 overexpression compromised Ldo45 function, resulting in cytosolic localization of Pdr16 (Fig. 4*B*). Thus, it seems likely that increased Pln1 or Tgl4 expression affected the stoichiometry of the seipin accessory factor subcomplex or the entire LD assembly complex to intervene LD assembly.

We further explored the impact of deleting each seipin accessory factor individually and in combination (Fig. 5*A*). Although only minor LD morphological changes were observed with each single mutant, we found enlarged and more aggregated LDs when mutations were combined, indicative of additive effects. Thus, seipin accessory factors, unlike seipin that is central to LD assembly, may primarily exert a regulatory role, such as controlling the flux of neutral lipid filling and LD protein and membrane lipid sorting. We envision that some seipin accessory factors may share redundant, but not identical, functions. For example, seipin accessory factors may play a role in defining the site for LD formation and/or participating in ER-LD contact site remodeling. The multifunctional enzyme Tgl4 is of particular interest as it is the only enzyme present in the LD assembly complex (23). Its catalytic activity has the potential to alter local diacylglycerol (DAG) levels or membrane phospholipids, such as phosphatidic acid, as to impact on protein-protein and/or protein-lipid binding. Pln1 is the yeast perilipin crucial for stabilizing the LD structure and is needed for efficient LD formation (21). Mammalian PLN3 has been implicated in LD biogenesis through binding to membranes enriched in DAG (24, 25). Whether yeast Pln1 is brought to the LD assembly complex through binding to DAG and whether the activity of Tgl4 contributes to the process merits additional investigation.

LDAF1 is the mammalian homolog of Ldo45 and has been implicated in defining the site of LD formation (12, 26). The LDAF1 protein harbors two hairpins necessary and sufficient for interacting with seipin’s hydrophobic domain as to facilitate LDs bulging out from the ER at low TAG concentration. It is possible that Ldo45 may exert a similar function as LDAF1. The fact that our two-hybrid interaction studies reveal interaction of Ldo45 with Ldb16 is consistent with the notion (Fig. 7*A*), given that Ldo45 binds seipin’s hydrophobic domain, which in yeast is provided by Ldb16 rather than Sei1 (11). In this study, we provide compelling evidence linking Ldo45 with Pln1 and Tgl4, suggesting that Ldo45 may work in conjunction with Pln1 and Tgl4 to assist LD budding through binding with Ldb16 in the seipin complex (Figs. 6 and 7). Pln1 and Tgl4 both have mammalian homologs (21, 27), although it remains unclear whether LD packaging in mammals operates in a similar fashion.

In this study, we observed that seipin complex and the seipin accessory factor subcomplex could form and associate together in the absence of neutral lipids (Fig. 6*D*). Moreover, we provide further evidence to support a dynamic rearrangement of these molecules during TAG filling, underscoring their orchestrated remodeling. The significance of these changes is not fully understood at present, but they might reflect conformational alterations associated with these molecules in initiating and driving LD assembly. To this end, our observation is also consistent with the existence of close and open conformations of seipin recently reported (28).

Collectively, the identification of seipin accessory factors lays the foundation for further understanding how neutral lipids, LD proteins, and lipids are sorted by the LD assembly machinery to shape LD formation. Further investigations are crucial to unveil the molecular mechanisms for how these factors involved in LD biogenesis. Identifying conserved features of these molecules with their putative mammalian counterparts will enhance our comprehension of their interactions with seipin at the ER-LD contact site for LD assembly.

## Experimental procedures

### Reagents

Monoclonal Anti-HA antibody (HA.C5) was purchased from Abcam (ab18181), peroxidase-anti-peroxidase (PAP-HRP) was from Jackson ImmunoResearch Laboratory, and Pierce High Sensitivity Strp-HRP (21130) was from Thermo Fisher Scientific. Homemade polyclonal anti-Sei1(Fld1) (19), anti-Ldb16 (29), anti-Pln1(Pet10) (29), anti-Ubx2 (29), anti-Erg6 (29), anti-Erg1 (29), and anti-Ole1 (30) were mentioned previously. Polyclonal anti-Ldo proteins antibody was raised against amino acids 74 to 87 and 132 to 146 of Ldo16. Polyclonal anti-Pah1 antibody was raised against amino acids 125 to 142, 322 to 340, and 757-755. Polyclonal anti-Tgl4 antibody was raised against amino acids 344 to 361, 447 to 464, and 855 to 872.

Various percentages of SurePAGE, Bis-Tris gels were purchased from GenScript, NuPAGE lithium dodecyl sulfate (LDS) sample buffer (4×) was from Thermo Fisher Scientific, 20× NuPAGE Mops SDS running buffer from Thermo Fisher Scientific, Immobilon Western Chemiluminescent HRP substrate from Merk Millipore, and Invitrogen BODIPY 493/503 from Thermo Fisher Scientific. Strp sepharose high performance was from Cytiva. cOmplete EDTA-free protease inhibitor cocktail tablets were from Roche.

### Yeast strains, plasmids, and growth conditions

All yeast strains, plasmids, and primers used in this study are listed in Table S1. We used a PCR-based transformation method for generating the yeast strains, and most of the yeast strains were made isogenic to the BY4742 (*MATα his3Δ1 leu2Δ0 lys2Δ0 ura3Δ0*) strain. Gene KO was performed by replacing the entire gene coding region with either the *Escherichia coli* Kanamycin resistance gene (*KAN*), *E. coli* hygromycin resistance gene (*HYG*), *Streptomyces* nourseothricin resistance gene (*cloNAT*), or the *Saccharomyces cerevisiae LEU2* gene. These genes were PCR-amplified from *pFA6a-KanMX6*, *pFA6a-HygMX6*, *pFA6a-NatMX6*, or *pFA6a-LEU2* vectors (31). Chromosomal tagging of the target gene at the 3′-end was achieved by integration of the TurboID-HA, PA (immunoglobulin G-binding domain), GFP, or mCherry tag genes using the *pFA6a-TurboID-HA-HIS3MX6*, *pFA6a-PA-HIS3MX6*, *pFA6a-GFP(S65T)-HIS3MX6*, *pFA6a-mCherry-KanMX6*, *pFA6a-mCherry-LEU*, and *pFA6a-mCherry-HygMX6* as template plasmids for PCR (31).

The plasmid *pFA6a-TurboID-HA-HIS3MX6* was generated by synthesizing TurboID-HA DNA fragment and ligating it into the PacI-AscI sites of the *pFA6a-GFP(S65T)-HIS3MX6*. *pRS426-LDO45* was generated by amplifying the *LDO45* promoter (675 bp) and ligating it into the SacI-BamHI site of *pRS416-CYC1* (32) to create *pRS416-P*_*LDO45*_*-T*_*CYCl*_. The *LDO45* ORF was synthesized and ligated into the BamHI-SalI sites of the *pRS416-P*_*LDO45*_*-T*_*CYCl*_ to make *pRS416-P*_*LDO45*_*-LDO45-T*_*CYCl*_. Finally, the SacI-KpnI digested fragment of the *P*_*LDO45*_*-LDO45-T*_*CYCl*_ was ligated with *pRS426* to create *pRS426-LDO45*. The plasmid *pRS416-P*_*LDO45*_*-LDO45-TurboID-HA-T*_*CYCl*_ was generated by amplifying the *LDO45* ORF and ligating it into the BamHI-EcoRI sites of the *pRS416-P*_*LDO45*_*-T*_*CYCl*_, followed by amplifying the TurboID-HA fragment and ligating it into the EcoRI-SalI sites. The plasmid *pRS416-LDO16* was created by amplifying *LDO16* promoter (400 bp) and *LDO16* ORF together and ligating it into the SacI-SalI sites of *pRS416-CYC1* to make *pRS416-LDO16*. The SacI-KpnI digested fragment of the *pRS416-LDO16*, containing the promoter, ORF of *LDO16*, and the terminator of *CYC1*, was ligated with *pRS426* to create *pRS426-LDO16*. The plasmid *pRS416-P*_*LDO16*_*-LDO16-TurboID-HA-T*_*CYCl*_ was generated by amplifying *LDO16* promoter (400 bp) and ORF and ligating it into the SacI-EcoRI sites of the *pRS416-CYC1*, followed by amplifying the TurboID-HA fragment and ligating it into the EcoRI-SalI sites. The plasmid *pRS426-TGL4* was created by amplifying *TGL4* promoter (600 bp), *TGL4* ORF, and *TGL4* terminator (250 bp) and ligating it into the BamHI-SalI sites of *pRS426*. The plasmid *pRS426-PLN1* was generated by amplifying entire *PLN1* promoter (300 bp), *PLN1* ORF, and *PLN1* terminator (300 bp) and ligating it into BamHI-XhoI sites of *pRS426*.

The plasmids *pGAD-SEI1*, *pGAD-LDO45*, *pGBD-SEI1*, *pGBD-LDB16*, *pGBD-LDO45*, *pGBD-LDB16*, and *pGBD-TGL4* were created by PCR amplification of the full-length genes and ligating them into the BamHI-SalI sites of the *pGAD-c1* or *pGBD-c1* (33). The plasmid *pGBD-PLN1* was created by PCR amplification of the full-length *PLN1* genes and ligating it into the BamHI-PstI sites of the *pGBD-c1*.

Yeast cells were grown in SC (synthetic complete) media (0.67% yeast nitrogen base, amino acids, and 2% glucose) at 30 °C unless otherwise mentioned.

### Fluorescence microscopy

Yeast cells were cultivated in SC medium starting from an initial *A*_600_ of 0.1 and grown for 24 h. Cultures were centrifuged and cells were resuspended in 50 mM Tris, pH 7.5 before visualization. Still images were captured by the Olympus IX81 fluorescence microscope equipped with a 100× Plan Apochromat oil immersion objective lens (NA = 1.4), a CMOS camera (ORCA-Flash 4.0; Hamamatsu Photonics), a GFP filter (488-nm excitation with a bandpass 500–530 nm), and a mCherry filter (561-nm excitation with a bandpass 575–630 nm). Image acquisition and processing were performed with MetaMorph (Molecular Devices). Images were cropped and adjusted for contrast and intensity by Photoshop (Adobe, https://www.adobe.com/products/) and placed on Illustrator (Adobe).

For LD imaging, cells were stained with 1 μg/ml BODIPY 494/503 for 20 min in the dark, followed by two washes with 50 mM Tris–HCl, pH 7.5. The cells were then resuspended in 50 mM Tris, pH 7.5 before visualization. We used MetaMorph manually count object tool to quantify clustered LDs. Stringent criteria were applied to the definition of clustered LDs, considering cells containing either more than eight LDs aggregated together or all LDs in the cell were clustered within approximately one-third of the cell volume, with the remainder of the cell devoid of LDs. To quantify cells containing supersized LDs, we specifically counted cells with fewer than two supersized LDs, irrespective of the presence of small aggregated LDs. Three independent images were subjected for quantification, with each image typically containing 200 to 300 (ranging from 183 to 466) counted cells.

### TurboID proximity labeling experiment

#### Strp-HRP blots

Yeast strains were cultured in SC medium containing complete amino acids and 2% glucose excluding biotin. The standard reaction involves the use of *arc1Δ* strain background. In these experiments, freshly grown cells at absorbance 0.35 were subjected for biotinylation by adding 50 mM biotin to a final concentration of 50 μM. The labeling process occurred at 30 °C and extended over a period of 6 h.

For sample preparation, 1 ml of culture was first mixed with 55 μl of 200 mM NaN_3_ to stop the reaction for 1 min. Subsequently, cells were centrifuged at 6200*g* for 30 s, and the resulting cell pellets were resuspended in 200 μl of 0.1 M NaOH for 5 min. Following centrifugation, cells were resuspended in 1× LDS sample buffer containing 5% β-mercaptoethanol (normalized to 100 μl buffer per 0.5 *A*_600_ cells). The samples were lysed by glass beads, heated to 55 °C for 10 min, and subjected to SDS-PAGE, followed by Western blot analysis using Strp-HRP. The same membrane blot was cut for the Western blotting procedure using other antibodies, such as anti-HA or peroxidase-anti-peroxidase, allowing for the comparison of protein sizes. Blots were developed using Immobilon Western Chemiluminescence HRP Substrate and captured using the UVP ChemiDoc-It imager and software (https://www.labortechnik.com/de/analysis-software-visionworksls).

#### Strp pull-down assay

Yeast strains were cultured in SC medium containing complete amino acids and 2% glucose, excluding biotin. Fresh cells were grown to log phase (*A*600 = 0.5–0.6), and biotinylation was initiated by adding 50 mM biotin to the culture to the final concentration of 50 μM. After 6 h, 40 *A*_600_ cells were harvested and subjected for spheroplasting. In brief, the cells were first incubated in ice-cold 1× PBS containing 20 mM NaN_3_ for 5 min. Following centrifugation, cells were resuspended in 100 mM Tris-SO_4_, pH9.4, 10 mM DTT, and incubated at 30 °C for 10 min. Subsequently, cells were resuspended in spheroplasting buffer containing 40 mM Hepes (pH 7.4), 1 M sorbitol, and crude lyticase at 30 °C for 30 min incubation. Spheroplasts were harvested by centrifugation at 900*g* for 5 min and stored immediately at −80 °C. Thawed spheroplasts were lysed in TurboID lysis buffer (50 mM Tris–HCl, pH 7.4, 500 mM NaCl, 0.4% SDS, 5 mM EDTA, 1% Triton X-100, and 1 mM DTT) containing 1× cOmplete EDTA-free protease inhibitor and 125 μM PMSF. After centrifugation at 13,000*g* for 10 min, a small portion of the resulting lysates was harvested, adjusted to 1× LDS sample buffer containing 5% β-mercaptoethaol and loaded as input. The rest of the lysates were incubated with 100 μl bed volume of Strp-sepharose beads, and the binding occurred at 4 °C for 4 h with gentle rotation. The beads were washed first with 2% SDS in 20 mM Tris–HCl (pH 7.4) containing 125 μM PMSF for two rounds, followed by five washes with TurboID lysis buffer containing 125 μM PMSF. Bound proteins were eluted with 150 μl of 25 mM biotin in 1× LDS sample buffer containing 5% β-mercaptoethanol. Samples were vortexed and heated to 95 °C for 5 min. After centrifugation, the supernatants were recovered and subjected to SDS-PAGE, followed by Western blot analysis using various antibodies. Blots were developed using Immobilon Western Chemiluminescence HRP Substrate and captured using the UVP ChemiDoc-It imager and software.

To conduct Strp pull-down experiments in an LD-induction system, yeast strains were cultivated in SC medium supplemented with complete amino acids, 2% raffinose, and 0.05% glucose, excluding biotin. A fresh culture of approximately 120 ml was prepared and grown to 0.5 absorbance, and the culture cells were divided into two. After washing with sterile ddH2O, one culture was resuspended in SC medium containing complete amino acids and 2% raffinose, excluding biotin, while the other culture was resuspended in SC medium containing complete amino acids and 2% galactose, excluding biotin. Biotinylation was initiated by adding 50 mM biotin to the cultures, reaching a final concentration of 50 μM, and the reaction was incubated at 30 °C for 3 h. Cells harvest, converting to spheroplasts, lysates preparation and Strp pull-down procedures were conducted as described above. Blots were developed using Immobilon Western Chemiluminescence HRP Substrate and captured using the UVP ChemiDoc-It imager and software.

### Yeast two-hybrid assay

The yeast strain PJ69-4a (33) was transformed with *pGAD-* and *pGBD-*containing plasmids and grown on SC-LEU-TRP plates. At least three independent transformants were assayed for their interactions on SC-LEU-TRP-HIS plates containing 1 mM 3-amimotriazole (3-AT). The plates were incubated at 30 °C for 3 to 8 days and photographed by the UVP ChemiDoc-It imager and software.

### Statistical analysis

All experiments were repeated for at least three times but only one representative result is shown. We used two-tailed Student *t* test (∗*p* < 0.05; ∗∗*p* < 0.01; ∗∗∗*p* < 0.001) for statistical analysis and the data are presented as mean ± SD

## Data availability

All data generated or analyzed during this study are included in this article or in the supplementary information files.

## Supporting information

This article contains supporting information.

## Conflict of interest

The authors declare that they have no conflicts of interest with the contents of this article.

## References

[1] Walther T.C., Chung J., Farese R.V. (2017). Lipid droplet biogenesis. Annu. Rev. Cell Dev. Biol..

[2] Fei W., Du X., Yang H. (2011). Seipin, adipogenesis and lipid droplets. Trends Endocrinol. Metab..

[3] Fei W., Shui G., Gaeta B., Du X., Kuerschner L., Li P. (2008). Fld1p, a functional homologue of human seipin, regulates the size of lipid droplets in yeast. J. Cell Biol..

[4] Liu L., Jiang Q., Wang X., Zhang Y., Lin R.C., Lam S.M. (2014). Adipose-specific knockout of SEIPIN/BSCL2 results in progressive lipodystrophy. Diabetes.

[5] Szymanski K.M., Binns D., Bartz R., Grishin N.V., Li W.P., Agarwal A.K. (2007). The lipodystrophy protein seipin is found at endoplasmic reticulum lipid droplet junctions and is important for droplet morphology. Proc. Natl. Acad. Sci. U. S. A..

[6] Tian Y., Bi J., Shui G., Liu Z., Xiang Y., Liu Y. (2011). Tissue-autonomous function of Drosophila seipin in preventing ectopic lipid droplet formation. PLoS Genet..

[7] Salo V.T., Li S., Vihinen H., Holtta-Vuori M., Szkalisity A., Horvath P. (2019). Seipin facilitates triglyceride flow to lipid droplet and counteracts droplet ripening *via* endoplasmic reticulum contact. Dev. Cell.

[8] Wang H., Becuwe M., Housden B.E., Chitraju C., Porras A.J., Graham M.M. (2016). Seipin is required for converting nascent to mature lipid droplets. Elife.

[9] Yan R., Qian H., Lukmantara I., Gao M., Du X., Yan N. (2018). Human SEIPIN binds anionic phospholipids. Dev. Cell.

[10] Sui X., Arlt H., Brock K.P., Lai Z.W., DiMaio F., Marks D.S. (2018). Cryo-electron microscopy structure of the lipid droplet-formation protein seipin. J. Cell Biol..

[11] Klug Y.A., Deme J.C., Corey R.A., Renne M.F., Stansfeld P.J., Lea S.M. (2021). Mechanism of lipid droplet formation by the yeast Sei1/Ldb16 Seipin complex. Nat. Commun..

[12] Chung J., Wu X., Lambert T.J., Lai Z.W., Walther T.C., Farese R.V. (2019). LDAF1 and seipin form a lipid droplet assembly complex. Dev. Cell.

[13] Eisenberg-Bord M., Mari M., Weill U., Rosenfeld-Gur E., Moldavski O., Castro I.G. (2018). Identification of seipin-linked factors that act as determinants of a lipid droplet subpopulation. J. Cell Biol..

[14] Teixeira V., Johnsen L., Martinez-Montanes F., Grippa A., Buxo L., Idrissi F.Z. (2018). Regulation of lipid droplets by metabolically controlled Ldo isoforms. J. Cell Biol..

[15] Wang S., Idrissi F.Z., Hermansson M., Grippa A., Ejsing C.S., Carvalho P. (2018). Seipin and the membrane-shaping protein Pex30 cooperate in organelle budding from the endoplasmic reticulum. Nat. Commun..

[16] Joshi A.S., Nebenfuehr B., Choudhary V., Satpute-Krishnan P., Levine T.P., Golden A. (2018). Lipid droplet and peroxisome biogenesis occur at the same ER subdomains. Nat. Commun..

[17] Choudhary V., Ojha N., Golden A., Prinz W.A. (2015). A conserved family of proteins facilitates nascent lipid droplet budding from the ER. J. Cell Biol..

[18] Choudhary V., El Atab O., Mizzon G., Prinz W.A., Schneiter R. (2020). Seipin and Nem1 establish discrete ER subdomains to initiate yeast lipid droplet biogenesis. J. Cell Biol..

[19] Wang C.W., Miao Y.H., Chang Y.S. (2014). Control of lipid droplet size in budding yeast requires the collaboration between Fld1 and Ldb16. J. Cell Sci..

[20] Klein I., Klug L., Schmidt C., Zandl M., Korber M., Daum G. (2014). Regulation of the yeast triacylglycerol lipases Tgl4p and Tgl5p by the presence/absence of nonpolar lipids. Mol. Biol. Cell.

[21] Gao Q., Binns D.D., Kinch L.N., Grishin N.V., Ortiz N., Chen X. (2017). Pet10p is a yeast perilipin that stabilizes lipid droplets and promotes their assembly. J. Cell Biol..

[22] Jacquier N., Choudhary V., Mari M., Toulmay A., Reggiori F., Schneiter R. (2011). Lipid droplets are functionally connected to the endoplasmic reticulum in Saccharomyces cerevisiae. J. Cell Sci..

[23] Rajakumari S., Daum G. (2010). Multiple functions as lipase, steryl ester hydrolase, phospholipase, and acyltransferase of Tgl4p from the yeast Saccharomyces cerevisiae. J. Biol. Chem..

[24] Khaddaj R., Stribny J., Cottier S., Schneiter R. (2023). Perilipin 3 promotes the formation of membrane domains enriched in diacylglycerol and lipid droplet biogenesis proteins. Front. Cell Dev. Biol..

[25] Choi Y.M., Ajjaji D., Fleming K.D., Borbat P.P., Jenkins M.L., Moeller B.E. (2023). Structural insights into perilipin 3 membrane association in response to diacylglycerol accumulation. Nat. Commun..

[26] Walther T.C., Kim S., Arlt H., Voth G.A., Farese R.V. (2023). Structure and function of lipid droplet assembly complexes. Curr. Opin. Struct. Biol..

[27] Kurat C.F., Wolinski H., Petschnigg J., Kaluarachchi S., Andrews B., Natter K. (2009). Cdk1/Cdc28-dependent activation of the major triacylglycerol lipase Tgl4 in yeast links lipolysis to cell-cycle progression. Mol. Cell.

[28] Arlt H., Sui X., Folger B., Adams C., Chen X., Remme R. (2022). Seipin forms a flexible cage at lipid droplet formation sites. Nat. Struct. Mol. Biol..

[29] Wang C.W., Lee S.C. (2012). The ubiquitin-like (UBX)-domain-containing protein Ubx2/Ubxd8 regulates lipid droplet homeostasis. J. Cell Sci..

[30] Huang L.J., Chen R.H. (2023). Lipid saturation induces degradation of squalene epoxidase for sterol homeostasis and cell survival. Life Sci. Alliance.

[31] Longtine M.S., McKenzie A., 3rd, Demarini D.J., Shah N.G., Wach A., Brachat A. (1998). Additional modules for versatile and economical PCR-based gene deletion and modification in Saccharomyces cerevisiae. Yeast.

[32] Mumberg D., Muller R., Funk M. (1995). Yeast vectors for the controlled expression of heterologous proteins in different genetic backgrounds. Gene.

[33] James P., Halladay J., Craig E.A. (1996). Genomic libraries and a host strain designed for highly efficient two-hybrid selection in yeast. Genetics.

