# Supplementary material for: The lipid droplet assembly complex consists of seipin and four accessory factors in budding yeast
Source: J Biol Chem. 2024 Jul 7;300(8):107534. doi: 10.1016/j.jbc.2024.107534 (PMC11342095; doi:10.1016/j.jbc.2024.107534)
Supplement: Supporting Figures [file mmc1.pdf]

Figure S1

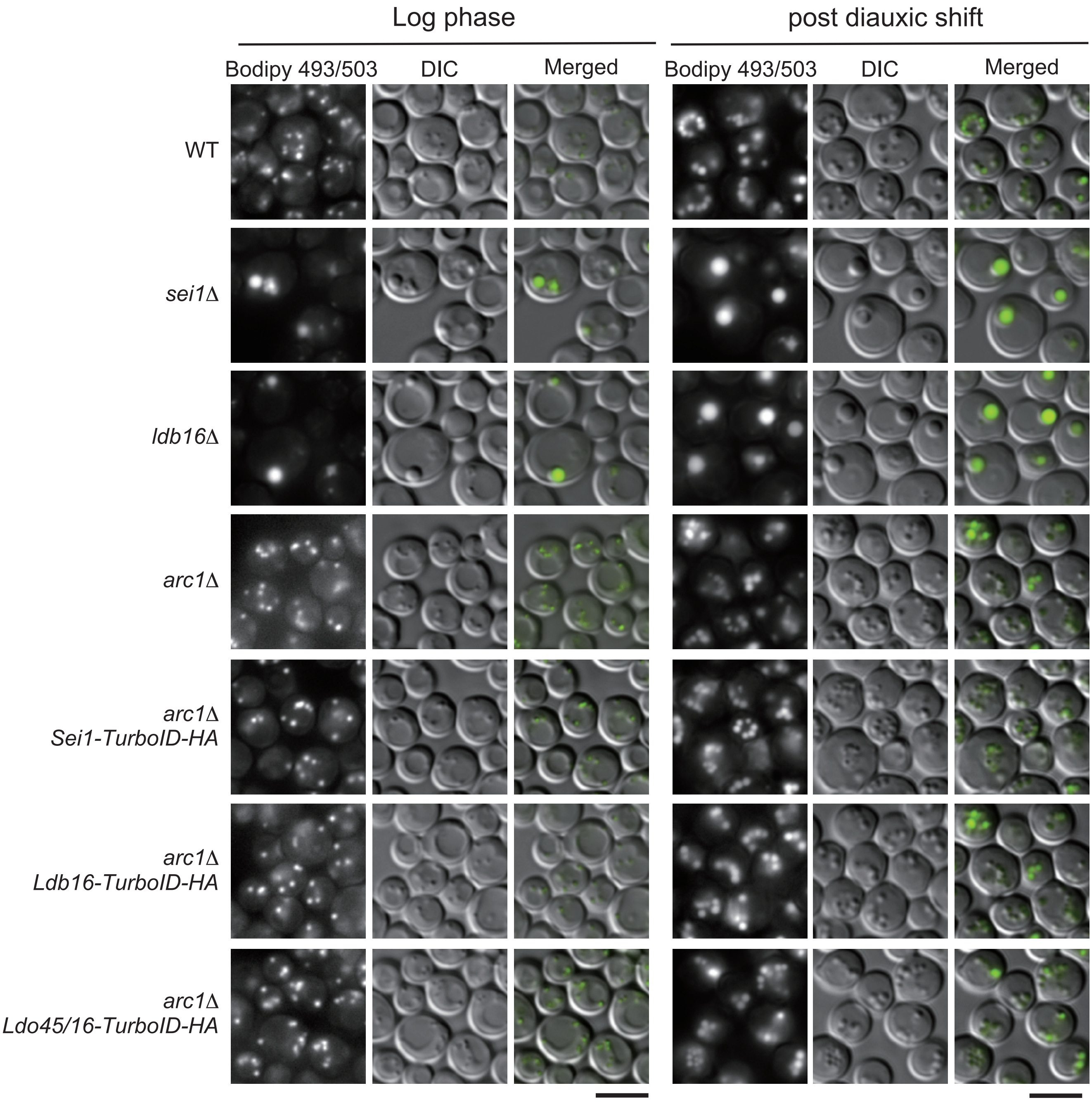

Figure S1: LD morphology in cells expressing Sei1, Ldb16, and Ldo45/16 tagged with TurboID-HA
Yeast strains CWY2522 (Wild-type), CWY3033(*sei1*Δ), CWT3385(*ldb16*Δ), CWY12910(*arc1*Δ), CWY12931 (*arc1*Δ *Sei1-TurboID-HA*), CWY12912 (*arc1*Δ *Ldb16-TurboID-HA*), and CWY12929 (*arc1*Δ *Ldo45/16-TurboID -HA*) were grown in SC-complete medium at 30C to log phase or for 24 h to post diauxic shift. Cells were stained with Bodipy 493/503 and imaged by Olympus IX81 fluorescence microscope. Scale bar, 5 μm.

Figure S2

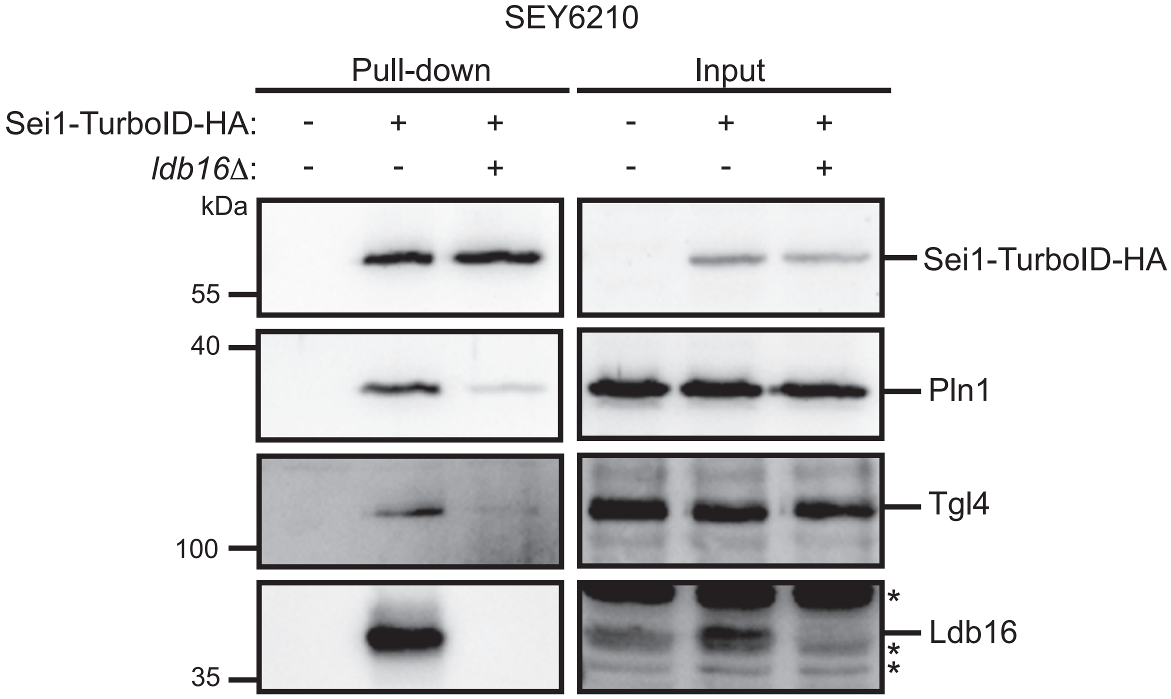

Figure S2: Sei1-TurboID-HA biotinylated Pln1 and Tgl4 proteins in the yeast strain SEY6210
Yeast strain CWY5989 (Wild-type), CWY11833 (Sei1-TurboID-HA), CWY11835 (*ldb16Δ* Sei1-TurboID-HA) were subjected for biotin labeling for 6 hours at 30C. Cells were harvested and converted to lysates with glass beads. The lysates (input) were subjected for Strp pull-down as described in the Experimental procedures. The input and pulled-down fractions were analyzed by Western blotting using an anti-HA antibody and antibodies against the indicated yeast proteins. *, antibody non-specific bands.

Figure S3

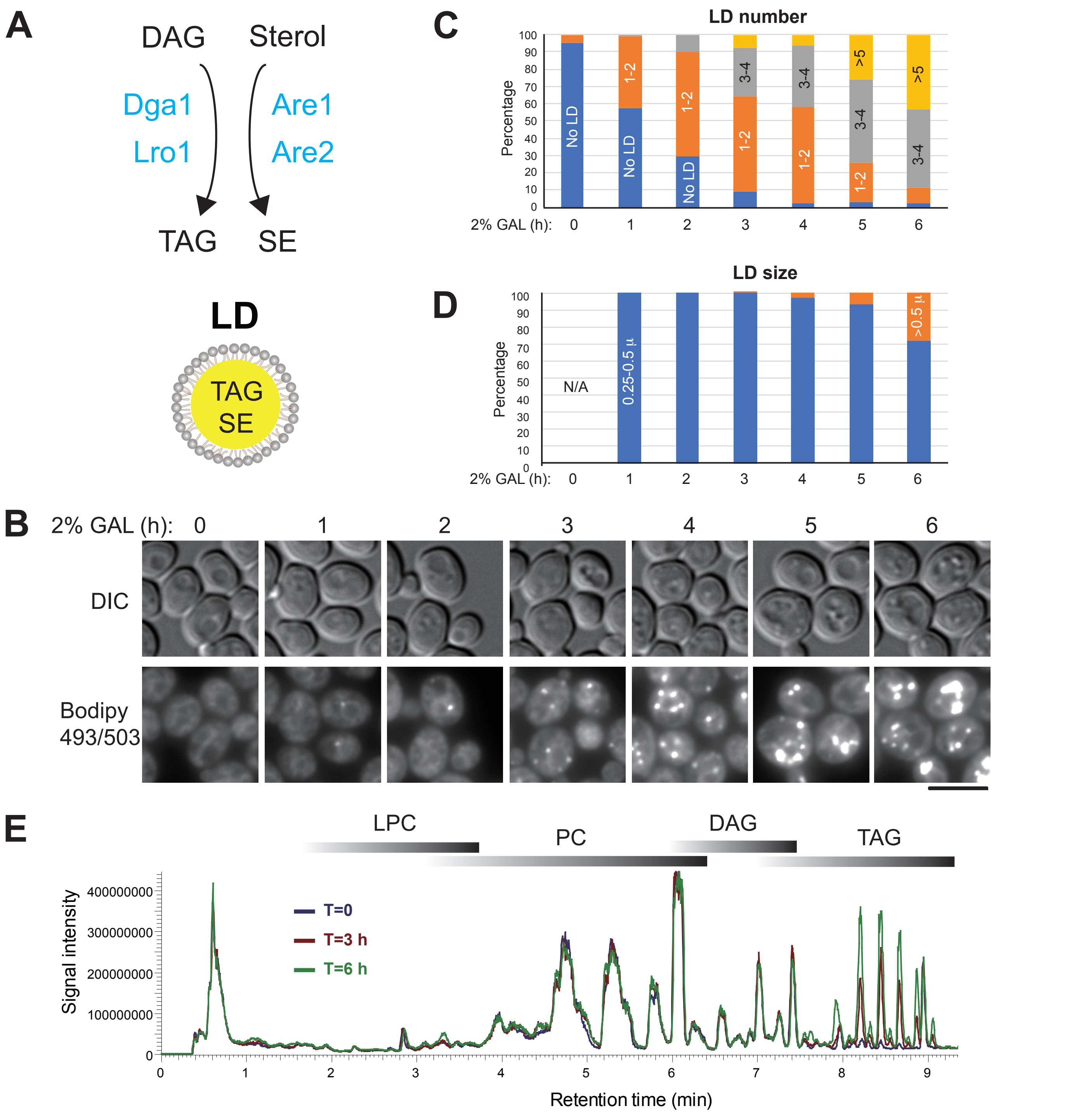

Figure S3: The LD-inducible yeast strain harboring Sei1-TurboID-HA triggered LD formation within 6 hours of galactose treatment

(A)The diagram of neutral lipid synthesis and storage in the yeast LD.

(B)The LD-inducible yeast strain CWY12322 harboring Sei1-TurboID-HA was shifted from medium containing raffinose to galactose at 30C. Portions of cells were collected at indicated time points for Bodipy 493/503 staining and imaged by Olympus IX81 fluorescence microscope. Scale bar, 5 μm.

(C)The number of LDs in cells shifted to medium containing galactose at different time points were quantified and plotted.

(D)The size of LDs in cells shifted to medium containing galactose at different time points were quantified and plotted.

(E)The yeast strain CWY12322 harboring Sei1-TurboID-HA was shifted from medium containing raffinose to galactose at 30C. Samples were collected at T=0, 3, and 6 hours after the shift and lipids were extracted. The chromatogram depicting the results of liquid chromatography-mass spectrometry analyses of lipids in the positive ion mode. LPC, lysophosphatidylcholine; PC, phosphatidylcholine; DAG, diacylglycerol; TAG, triacylglycerol.
