# Supplementary material for: The lipid droplet assembly complex consists of seipin and four accessory factors in budding yeast
Source: J Biol Chem. 2024 Jul 7;300(8):107534. doi: 10.1016/j.jbc.2024.107534 (PMC11342095; doi:10.1016/j.jbc.2024.107534)
Supplement: Supporting tables [file mmc2.pdf]

Table S1: Yeast strains, plasmids, and primers used in this study

(1) Strain

Strain	Description	Source
**BY4742**	*MATα his3Δ1 leu2Δ0 lys2Δ0 ura3Δ0*	Lab collection
**CWY12910**	*arc1Δ::LEU BY4742*	This study
**CWY12931**	*Sei1-TurboID-HA::HIS arc1Δ::LEU BY4742*	This study
**CWY12912**	*Ldb16-TurboID-HA::HIS arc1Δ::LEU BY4742*	This study
**CWY12991**	*Ldb16-PA::KAN Sei1-TurboID-HA::HIS arc1Δ::LEU BY4742*	This study
**CWY12993**	*Ldo45/16-PA::KAN Sei1-TurboID-HA::HIS arc1Δ::LEU BY4742*	This study
**CWY12975**	*ldb16Δ::KAN Sei1-TurboID-HA::HIS arc1Δ::LEU BY4742*	This study
**CWY12948**	*Ldo45/16Δ::KAN Sei1-TurboID-HA::HIS arc1Δ::LEU BY4742*	This study
**CWY12986**	*Sei1-PA::KAN Ldb16-TurboID-HA::HIS arc1Δ::LEU BY4742*	This study
**CWY12989**	*Ldo45/16-PA::KAN Ldb16-TurboID-HA::HIS arc1Δ::LEU BY4742*	This study
**CWY12951**	*sei1Δ::KAN Ldb16-TurboID-HA::HIS arc1Δ::LEU BY4742*	This study
**CWY12954**	*Ldo45/16Δ::KAN Ldb16-TurboID-HA::HIS arc1Δ::LEU BY4742*	This study
**CWY12929**	*Ldo45/16-TurboID-HA::HIS arc1Δ::LEU BY4742*	This study
**CWY13037**	*Tgl4-PA::KAN Ldo45/16-TurboID-HA::HIS arc1Δ::LEU BY4742*	This study
**CWY13079**	*Tgl5-PA::KAN Ldo45/16-TurboID-HA::HIS arc1Δ::LEU BY4742*	This study
**CWY12990**	*Pln1-PA::KAN Ldo45/16-TurboID-HA::HIS arc1Δ::LEU BY4742*	This study
**CWY13040**	*Erg6-PA::KAN Ldo45/16-TurboID-HA::HIS arc1Δ::LEU BY4742*	This study
**CWY13083**	*Pex30-PA::KAN Ldo45/16-TurboID-HA::HIS arc1Δ::LEU BY4742*	This study
**CWY13013**	*ldo45/16Δ::cloNAT arc1Δ::LEU BY4742*	This study
**CWY2989**	*Erg6-mCherry::KAN BY4742*	This study
**CWY2983**	*Tgl4-mCherry::KAN BY4742*	This study
**CWY3145**	*Pln1-GFP::HIS Erg6-mCherry::KAN BY4742*	This study
**CWY3127**	*Tgl4-GFP::HIS Erg6-mCherry::KAN BY4742*	This study

**CWY3223**	*Tgl4-GFP::HIS Tgl5-mCherry::KAN BY4742*	This study
**CWY13231**	*Tgl4-GFP::HIS Sei1-mCherry::LEU BY4742*	This study
**CWY5064**	*Ldb16-GFP::HIS Sei1-mCherry::HYG BY4742*	This study
**CWY13232**	*Pln1-GFP::HIS Sei1-mCherry::HYG BY4742*	This study
**CWY3240**	*sei1Δ::HYG Pln1-GFP::HIS Erg6-mCherry::KAN BY4742*	This study
**CWY3234**	*sei1Δ::HYG Tgl4-GFP::HIS Erg6-mCherry::KAN BY4742*	This study
**CWY13196**	*ldo45/16Δ::HYG Tgl4-GFP::HIS Erg6-mCherry::KAN BY4742*	This study
**CWY13255**	*ldb16Δ::HYG Pln1-GFP::HIS Sei1-mCherry::HYG BY4742*	This study
**CWY13249**	*ldb16Δ::HYG Tgl4-GFP::HIS Sei1-mCherry::HYG BY4742*	This study
**CWY3048**	*pln1Δ::KAN BY4742*	This study
**CWY13017**	*ldo45/16Δ::cloNAT BY4742*	This study
**CWY9415**	*tgl4Δ::KAN BY4742*	This study
**CWY13109**	*ldo45/16Δ::cloNAT tgl4Δ::KAN BY4742*	This study
**CWY13162**	*ldo45/16Δ::HYG pln1Δ::cloNAT tgl4Δ::KAN BY4742*	This study
**CWY13151**	*ldo45/16Δ::HYG pln1Δ::KAN BY4742*	This study
**CWY13118**	*pln1Δ::cloNAT tgl4Δ::KAN BY4742*	This study
**CWY5802**	*Pdr16-GFP::LEU Erg6-mCherry::KAN BY4742*	This study
**CWY13049**	*tgl4Δ::KAN Ldo45/16-TurboID-HA::HIS arc1Δ::LEU BY4742*	This study
**CWY13076**	*pln1Δ::KAN Ldo45/16-TurboID-HA::HIS arc1Δ::LEU BY4742*	This study
**CWY12957**	*sei1Δ::KAN Ldo45/16-TurboID-HA::HIS arc1Δ::LEU BY4742*	This study
**CWY12960**	*ldb16Δ::KAN Ldo45/16-TurboID-HA::HIS arc1Δ::LEU BY4742*	This study
**CWY13025**	*pex30Δ::KAN Ldo45/16-TurboID-HA::HIS arc1Δ::LEU BY4742*	This study
**CWY3033**	*sei1Δ::KAN BY4742*	(Wang *et al.*,2014)
**CWY13007**	*Ldo45/16-TurboID-HA::HIS* P_{GAL1}*-Lro1::KAN are1Δ are2Δ dga1Δ BY4742*	This study
**CWY13264**	*Ldo45/16-TurboID-HA::HIS lro1Δ::cloNAT are1Δ are2Δ dga1Δ BY4742*	This study
**CWY12322**	*Sei1-TurboID-HA::HIS* P_{GAL1}*-Lro1::KAN are1Δ are2Δ dga1Δ BY4742*	This study
**CWY13167**	*Sei1-TurboID-HA::HIS lro1Δ::cloNAT are1Δ are2Δ dga1Δ BY4742*	This study

**CWY12529**	*Ldb16-TurboID-HA::HIS P_{GAL1}-Lro1::KAN are1Δ are2Δ dga1Δ BY4742*	This study
**CWY13102**	*Ldb16-TurboID-HA::HIS lro1Δ::cloNAT are1Δ are2Δ dga1Δ BY4742*	This study
**CWY13202**	*ldo45/16Δ::HYG Pdr16-GFP::LEU Erg6-mCherry::KAN BY4742*	This study
**CWY13200**	*ldo45/16Δ::HYG Pln1-GFP::HIS Erg6-mCherry::KAN BY4742*	This study
**CWY13208**	*ldo45/16Δ::HYG Tgl4-GFP::HIS Erg6-mCherry::KAN BY4742*	This study
**CWY13179**	*sei1Δ::HIS Pdr16-GFP::LEU Erg6-mCherry::KAN BY4742*	This study
**CWY13202**	*ldo45/16Δ::HYG Pdr16-GFP::LEU Erg6-mCherry::KAN BY4742*	This study
**CWY13187**	*pln1Δ::cloNAT Pdr16-GFP::LEU Erg6-mCherry::KAN BY4742*	This study
**CWY13191**	*tgl4Δ::HYG Pdr16-GFP::LEU Erg6-mCherry::KAN BY4742*	This study
**PJ69-4a**	*MATa, trp1-901, leu2-3,112, ura3-52, his3-200, gal4Δ, gal80Δ, GAL2-ADE2, LYS2::GAL1-ADE2, LYS2::GAL1-HIS3, met2::GAL7-lacZ*	(James et al., 1996)
**CWY13260**	*sei1Δ::LEU ldoΔ::HYG Erg6-mCherry::KAN BY4742*	This study
**CWY5859**	*sei1Δ::HYG P_{Sei1}-SEI1-T_{Cyc1}::URA SEY6210*	This study
**CWY11833**	*sei1Δ::HYG P_{Sei1}-SEI1-TurboID-HA-T_{Cyc1}::URA SEY6210*	This study
**CWY11835**	*ldb16Δ::HIS sei1Δ::HYG P_{Sei1}-SEI1-TurboID-HA-T_{Cyc1}::URA SEY6210*	This study

(2) Plasmids

Name	Description
pRS416-P_{LDO45}-LDO45-TurboID-HA-T_{CYC1}	*LDO45 promoter-LDO45 ORF-TurboID-HA-CYC1 terminator* cloned into *pRS416*
pRS416-P_{LDO16}-LDO16-TurboID-HA-T_{CYC1}	*LDO16 promoter-LDO16 ORF-TurboID-HA-CYC1 terminator* cloned into *pRS416*
pRS426	YE-type shuttle vector, a cloning phasmid containing URA3 auxotrophic marker

pRS426-SEI1	*SEI1 ORF* plus 300 bp upstream and 300 bp downstream sequence cloned into *pRS426*
pRS426-LDB16	*LDB16 ORF* plus 300 bp upstream and 300 bp downstream sequence cloned into *pRS426*
pRS426-LDO45	*LDO45 promoter(675bp)-LDO45 ORF-CYC1 terminator* cloned into *pRS426*
pRS426-LDO16	*LDO16 promoter(400bp)-LDO16 ORF-CYC1 terminator* cloned into *pRS426*
pRS426-TGL4	*TGL4 ORF* plus 600 bp upstream and 250 bp downstream sequence cloned into *pRS426*
pRS426-PLN1	*PLN1 ORF* plus 300 bp upstream and 300 bp downstream sequence cloned into *pRS426*
pFA6a-TurboID-HA-HIS3MX6	*TurboID-HA* cloned into *pFA6a-3HA-HisMX6*
pRS416-P_{CYC1}-GFP-LDO45-T_{CYC1}	*CYC1 promoter-GFP-LDO45 ORF -CYC1 terminator* cloned into *pRS416*
pRS416-P_{CYC1}-GFP-LDO16-T_{CYC1}	*CYC1 promoter-GFP-LDO16 ORF-CYC1 terminator* cloned into *pRS416*
pRS416-Sec63-mCherry	*SEC63 promoter and ORF-mCherry-CYC1 terminator cloned into pRS416*
pUC57-Ldo45 ORF	Synthesized LDO45 ORF sequence:<br>ATGGCTGCAAGAAACAGAAGAAAGAATAATAAGAAGAAATCTTTGTT<br>AGTTACTTCTGCTGCACAAGAAAAGAATGCTACATACGTTTTGGTTGC<br>AGAAGAATTGCATAAGAAAACTATCGATTTGAACATGGGTACTGAAA<br>CACCATTGACAGAAAATCATGAAAATCCAATTCCAGCTAAGGAGTTTA<br>AACATCAACAAAAGTTGGAACCAATTGATGAACATGATGATGGTGAA<br>GATGAATTGTCTATTAAGTTTAAATCTATGACTAAATCTTCAGGTCCAA<br>TCACAGAAGCTGAAGTTCAAAAGTTGTTGTTGTCATACGCTTTTACTTC<br>TGCTGCAATCCAAGAAGATGAAAACGAAAAGGAATCTAGACATTACC<br>CAATTAAACCACCATCTCCATCAGCTTCTTCATTATCAGCATATTTTCA<br>ATCTTTTGTTGAAAAGTGTAAGCAAGTTTTCTATAACTTCTCTTTGCAA<br>ACTGTTGAAAAATTAAATGCTTTGCAAAATTCATTATATGAAGTTTTCT<br>GGATCATTTTTATATATTTGAACTACTGGTTTCCAAATGTTGGTGACTA<br>CGTTTCTAATACATTTGGTCAACAAGATTCAATCATCATCAGAATCTCT<br>TTGTCAAAGTCTCATTTCAGAGCTTTGAGAGAAAAATCTTCACAAAAG

	GTTCAACAAGCAGTTAAAAATATCTATTTCTGTTTCCAAGAAAAGCCA<br>TACTTAACTGCTTTTAAAGTTTCTTTCGCAATCGGTTTAGTTATTCCAT<br>GTTCTTTGTTGTTTTTGATTATGGTTTCAACTGCTACTTTCTTTTTCTTTG<br>TTTATTTGACATTGTTTGTTGTTATTGGTTTCTTTTCTTCATTGTTTATTA<br>TTCCATTGTTGGGTATCTCTTTCGTTTTCGCTATCGGTGTTGTTTCATTC<br>GGTTTCTGTTCAAACATGTCTTTTAAAATGGCACAATTGATCTATGTTA<br>GAGCTGATGCATTTTTGAAGAAAGTTTTGGATAAGATGGCTTTGCAAA<br>CACAACCAGCACAATTGCAAGAACCACAAGAACCATTATCAACTTTGA<br>GACCAGTTTCTAATCCAACAATTCCATCACCATTGAGACAAACTGCTA<br>GACCATCTAAGTTCGTTACAGAAGAAGATGTTATCTTTGAACCAGTTT<br>CAGCACAATCTGCTATTGCAAGATCTTTAGAAACTACAGCTAATAAGG<br>CAGGTAATAAGTTTCAATTGTCATAA<br>cloned into *pUC57*
pUC57-TurboID-HA	Synthesized TurboID-HA sequence:<br>ATGAAGGATAACACTGTTCCATTGAAGTTGATCGCTTTGTTGGCAAAT<br>GGTGAATTTCATTCTGGTGAACAATTAGGTGAAACATTGGGCATGTCA<br>AGAGCTGCTATTAATAAGCATATCCAAACTTTGAGAGATTGGGGTGTT<br>GATGTTTTTACAGTTCCTGGTAAAGGTTATTCTTTACCAGAACCAATCC<br>CATTGTTGAACGCTAAGCAAATCTTAGGTCAATTGGATGGTGGTTCTG<br>TTGCAGTTTTGCCAGTTGTTGATTCAACTAACCAATATTTGTTGGATAG<br>AATCGGTGAATTGAAGTCTGGTGACGCTTGTATTGCAGAATACCAACA<br>AGCTGGTAGAGGTTCTAGAGGTAGAAAATGGTTTTCACCATTCGGTGC<br>AAATTTGTATTTGTCAATGTTCTGGAGATTGAAGAGAGGTCCAGCTGC<br>AATTGGTTTAGGTCCAGTTATTGGTATCGTTATGGCTGAAGCATTGAG<br>AAAGTTGGGTGCTGATAAAGTTAGAGTCAAGTGGCCAAACGATTTGTA<br>CTTGCAAGATAGAAAGTTGGCTGGTATCTTGGTTGAATTGGCAGGTAT<br>TACTGGTGACGCTGCACAAATTGTTATTGGTGCTGGTATTAATGTTGCA<br>ATGAGAAGAGTTGAAGAATCTGTTGTTAATCAAGGTTGGATTACATTG<br>CAAGAAGCTGGTATTAATTTGGATAGAAACACTTTGGCTGCAACATTG<br>ATCAGAGAATTAAGAGCTGCATTAGAATTGTTTGAACAAGAAGGTTTG

	GCACCATATTTGCCAAGATGGGAAAAATTGGATAACTTCATCAACAGA<br>CCTGTTAAGTTGATCATCGGTGACAAGGAAATCTTCGGTATCTCAAGA<br>GGTATTGATAAACAAGGTGCTTTGTTGTTGGAACAAGATGGTGTTATT<br>AAACCATGGATGGGTGGTGAAATCTCTTTGAGATCAGCTGAAAAGGC<br>ATATCCATACGATGTTCCAGATTACGCTTAA<br>cloned into *pUC57*
pGAD-SEI1	*SEI1 ORF* cloned into *pGAD-c1*
pGAD-LDO45	*LDO45 ORF* cloned into *pGAD-c1*
pGBD-LDB16	*LDB16 ORF* cloned into *pGAD-c1*
pGBD-LDO16	*LDO16 ORF* cloned into *pGBD-c1*
pGBD-PLN1	*PLN1 ORF* cloned into *pGBD-c1*
pGBD-TGL4	*TGL4 ORF* cloned into *pGBD-c1*
pGAD-c1	(James et al., 1996)
pGBD-c1	(James et al., 1996)

(3) Primers

**Name**	**Sequence**
5’_TurboID_HA_PR_PacI	AAAAATTAATTAACATGAAGGATAACACTGTTC
3’_TurboID_HA_PR_AscI	AAAAAGGCGCGCCTTAAGCGTAATCTGGAACATC
5'_Sei1_PA	TTTTGTTAGAAAGGGTCAGGAAAAATCCAAGAAACATAGCcggatccccgggttaattaa
3'_Sei1_PA	TTCAGGGCGAAGAAGCAGAATGCTGGAAGAGCCTACTTACgaattcgagctcgtttaaac
5'_Ldb16_PA	GGACATCGTTAATATAAAGATTTTACGAAGGAATTCTAGGcggatccccgggttaattaa
3'_Ldb16_PA	TCTATCATTCACTTGTTAGTGCATGAGAAGAAGTAATTGCgaattcgagctcgtttaaac
5'_Sei1_KO	TCAAGAAAATAAGATAAAGTGAATAGGAAGGATGAAAATCcggatccccgggttaattaa

5'_KO_Ldb16	GGAGGAGAAAGCAGGTATATAACTAGCCGCAATATGTTTGcggatccccgggttaattaa
5_ldo16_PA	GACTACTGCTAATAAAGCGGGTAATAAGTTCCAGCTCTCTcggatccccgggttaattaa
3_ldo16_PA	AGGCCCATATGTAATAAAAAACTGAGTGTAATTTATAAGGgaattcgagctcgtttaaac
5_Ldo45_KO	GTGACATCTGAAAAACATCCAATACTCCGATGGCAGCCAGcggatccccgggttaattaa
5'_Tgl4_PA	TTCTTCAACGCAGCACAAAAGCACCACCAGTTTTACTCAAcggatccccgggttaattaa
3'_Tgl4_PA	TTTCCGCTTTGAAAATGATATGAATGAGAAAGGCCATTCGgaattcgagctcgtttaaac
5'_Pln1_PA	AGACCAAACCAATTCTAAGCCCGCGGCTGTGTCGACCAATcggatccccgggttaattaa
3'_Pln1_PA	ATAATAGAATAATAACAAATAACTATATAAGAGTGGCAGGgaattcgagctcgtttaaac
5'_Tgl5_PA	AAACGACAATTTCATGAACAATTCAGACATTTTTCAAAATcggatccccgggttaattaa
3'_Tgl5_PA	ATATAACAAAATGGCATGTACCTTTTGGAGTTGATGGAGGgaattcgagctcgtttaaac
5'_Erg6_PA	CGCCGAAACCCCCTCCCAAACTTCCCAAGAAGCAACTCAAcggatccccgggttaattaa
3'_Erg6_PA	TTATCTGCATATATAGGAAAATAGGTATATATCGTGCGCTgaattcgagctcgtttaaaca
5'_Pex30_PA	TCAAATCCAACCATTGGTCGCGATAGCAAGAAGGCCGTAcggatccccgggttaattaa
3'_Pex30_PA	TAGAGATTATATTATGTAAAGGTAAAAACGGGAGCGAGCAgaattcgagctcgtttaaaca
5'_Pdr16_PA	AAAACTTCTTTACCCAGTAAAATCGGAAAGCAGTACCGTGcggatccccgggttaattaa
3'_Pdr16_PA	TTTATTTATTATATATTATAGTGCATTATCATTATCTATCgaattcgagctcgtttaaac
5'_KO_Pdr16	CTTTATATAAAAAAAATTACAAAAGCAAAAAATGTTCAAGcggatccccgggttaattaa
5_Tgl4_KO	GGGAGTACAGGTATATGTAATAAAAGTCTGAATGAGCAGCcggatccccgggttaattaa
5'_KO_Pet10	CACTCACTCTCCAGAACCGTAGCGTAATTATGTCTGAATCcggatccccgggttaattaa
5_Pex30_KO	GATCCTCCGGAGTGTAAAAACTGATTTTCAATGAGTGGTAcggatccccgggttaattaa
3_Pex30_PA	TAGAGATTATATTATGTAAAGGTAAAAACGGGAGCGAGCAgaattcgagctcgtttaaac
5’_Ldo45_P675_SacI	AAAAAGAGCTCGTCTTCTCTCCTGGCCAGTTATC
3’_Ldo45_P675_BamHI	AAAAAGGATCCCGGAGTATTGGATGTTTTTCAGATG
5’_Ldo45_BamHI	AAAAGGATCCATGGCTGCAAGAAAC
3’_Ldo45_STOP_SalI	AAAAAGTCGACTTATGACAATTGAAACTTATTACC
5’_Ldo16_P400_SacI	AAAAAGAGCTCCTCGACCTTAGCGCGGCTAATC
3’_Ldo16_STOP_SalI	AAAAAGTCGACTTAAGAGAGCTGGAACTTATTACC

3’_Ldo16_EcoRI	AAAAAGAATTCAGAGAGCTGGAACTTATTACC
3'_Ldo45_EcoRI	AAAAAGAATTCTGACAATTGAAACTTATTACC
5’_Sei1_SacI_300	AAAAAGAGCTCAGTTGACAACACCAAGCC
3’_Sei1_BamHI_300	AAAAAGGATCCGACCATGTGTCTTTTGCC
5'_Ldb16_300_BamHI	AAAAAGGATCCTTGCATGCGCAAGTTGGT
3'_Ldb16_300_SalI	AAAAAGTCGACCCTGAGTATGCTATTAGC
5'_Tgl4_300_BamHI	AAAAGGATCCCGCTTCAAAAAGTCATTAGG
3'_Tgl4_300_SalI	AAAAGTCGACTTGTCAAAGTCTTCGAAAT
5'_Pln1_300_BamHI	AAAAGGATCCTCGCAAGAGTGGTCATAACC
3'_Pln1_300_XhoI	AAAACTCGAGGAAAGTTTGTTTCTTCTTCC
